# Supplementary material for: Assessing adherence to physical activity guidelines and correlates among older Korean adults with a focus on 10-minute bout duration using subjective and objective measures
Source: PLoS One. 2025 Jun 9;20(6):e0324342. doi: 10.1371/journal.pone.0324342 (PMC12148133; doi:10.1371/journal.pone.0324342)
Supplement: S1 Table — OR, odds ratio; CI, confidence interval; HDL, high-density lipoprotein. (DOCX) [file pone.0324342.s001.docx]

**S1 Table.** The odds ratios [95% CI] of participant characteristics associated with achieving ≥150 minutes per week of MVPA guidelines measured by accelerometer (>1952 cpm, ≥10 minute bouts)

|  | **Model 1^a^** | | **Model 2^b^** | | **Model 3^c^** | |
| --- | --- | --- | --- | --- | --- | --- |
|  | **OR [95% CI]** | **p-value** | **OR [95% CI]** | **p-value** | **OR [95% CI]** | **p-value** |
| **Sex, male** | 2.88 [1.66, 5.09] | **<0.001** | 3.38 [1.21, 9.49] | **0.020** | 2.30 [1.31, 4.11] | **0.004** |
| **Age** | 0.93 [0.87, 0.98] | **0.013** | 0.93 [0.86, 0.99] | **0.034** |  |  |
| **Education** | 1.10 [0.86, 1.41] | 0.445 | 0.98 [0.74, 1.29] | 0.864 |  |  |
| **Standardized household income** | 1.09 [0.84, 1.39] | 0.493 | 1.00 [0.75, 1.30] | 0.986 |  |  |
| **Economic activity** | 0.39 [0.20, 0.72] | **0.004** | 0.42 [0.21, 0.80] | **0.010** | 0.48 [0.25, 0.88] | **0.022** |
| **Marital status** | 0.89 [0.42, 1.92] | 0.750 | 0.93 [0.41, 2.16] | 0.857 |  |  |
| **Lifetime smoking: 5 or more packs** | 0.84 [0.40, 1.82] | 0.650 | 0.84 [0.37, 1.91] | 0.666 |  |  |
| **Drinking alcohol** | 0.95 [0.52, 1.71] | 0.861 | 0.91 [0.47, 1.75] | 0.781 |  |  |
| **Body mass index** | 0.99 [0.90, 1.08] | 0.744 | 1.01 [0.91, 1.12] | 0.814 |  |  |
| **Hypertension** | 0.69 [0.40, 1.19] | 0.185 | 0.69 [0.37, 1.26] | 0.227 |  |  |
| **Diabetes** | 0.82 [0.42, 1.52] | 0.545 | 1.04 [0.50, 2.09] | 0.909 |  |  |
| **Hypercholesterolemia** | 1.47 [0.84, 2.58] | 0.174 | 1.49 [0.79, 2.82] | 0.217 |  |  |
| **Hypertriglyceridemia** | 0.08 [0.00, 0.40] | **0.015** | 0.11 [0.01, 0.55] | **0.034** | 0.11 [0.01, 0.52] | **0.030** |
| **Low HDL cholesterol** | 0.36 [0.17, 0.68] | **0.003** | 0.38 [0.18, 0.76] | **0.008** | 0.36 [0.17, 0.68] | **0.003** |
| **Unmet medical needs** | 0.31 [0.05, 1.09] | 0.121 | 0.31 [0.05, 1.13] | 0.127 | 0.31 [0.05, 1.09] | 0.119 |
| **Sustained depression for more than 2 weeks** | 0.74 [0.26, 1.76] | 0.520 | 0.69 [0.24, 1.72] | 0.457 |  |  |
| **Activity limitations** | 0.93 [0.39, 2.01] | 0.862 | 0.99 [0.37, 2.40] | 0.984 |  |  |

OR, odds ratio; CI, confidence interval; HDL, high-density lipoprotein

^a^Model 1 includes age, sex, and activity limitations as covariates, along with each variable in the column being added one at a time.

^b^Model 2 includes all the variables simultaneously to examine their combined effects on the outcome.

^c^Model 3 uses a two-step lasso: first selecting main effects, then considering interaction terms if warranted.
