# Supplementary material for: Assessing adherence to physical activity guidelines and correlates among older Korean adults with a focus on 10-minute bout duration using subjective and objective measures
Source: PLoS One. 2025 Jun 9;20(6):e0324342. doi: 10.1371/journal.pone.0324342 (PMC12148133; doi:10.1371/journal.pone.0324342)
Supplement: S2 Table — OR, odds ratio; CI, confidence interval; HDL, high-density lipoprotein. (DOCX) [file pone.0324342.s002.docx]

**S2 Table.** The odds ratios [95% CI] of participant characteristics associated with achieving ≥150 minutes per week of MVPA guidelines measured by accelerometer (>1040 cpm, ≥10 minute bouts with 1~2 minute tolerance)

|  | **Model 1^a^** | | **Model 2^b^** | | **Model 3^c^** | |
| --- | --- | --- | --- | --- | --- | --- |
|  | **OR [95% CI]** | **p-value** | **OR [95% CI]** | **p-value** | **OR [95% CI]** | **p-value** |
| **Sex, male** | 2.55 [1.67, 3.93] | **<0.001** | 1.81 [0.82, 4.14] | 0.148 | 1.45 [0.90, 2.34] | 0.121 |
| **Age** | 0.90 [0.86, 0.94] | **<0.001** | 0.91 [0.86, 0.95] | **<0.001** |  |  |
| **Education** | 0.96 [0.79, 1.18] | 0.717 | 0.90 [0.72, 1.13] | 0.366 |  |  |
| **Standardized household income** | 0.98 [0.79, 1.24] | 0.884 | 0.94 [0.75, 1.21] | 0.632 |  |  |
| **Economic activity** | 0.98 [0.63, 1.53] | 0.917 | 0.96 [0.59, 1.55] | 0.862 |  |  |
| **Marital status** | 1.56 [0.94, 2.59] | 0.086 | 1.58 [0.91, 2.74] | 0.101 | 1.89 [1.15, 3.12] | **0.013** |
| **Lifetime smoking: 5 or more packs** | 1.20 [0.60, 2.36] | 0.594 | 1.31 [0.64, 2.65] | 0.450 |  |  |
| **Drinking alcohol** | 0.90 [0.56, 1.43] | 0.643 | 0.92 [0.56, 1.50] | 0.738 |  |  |
| **Body mass index** | 0.97 [0.90, 1.03] | 0.304 | 0.98 [0.91, 1.06] | 0.681 |  |  |
| **Hypertension** | 0.77 [0.49, 1.19] | 0.232 | 0.87 [0.53, 1.41] | 0.560 |  |  |
| **Diabetes** | 0.45 [0.28, 0.72] | **0.001** | 0.49 [0.30, 0.81] | **0.005** | 0.49 [0.31, 0.78] | **0.003** |
| **Hypercholesterolemia** | 1.11 [0.72, 1.74] | 0.632 | 1.40 [0.86, 2.30] | 0.184 |  |  |
| **Hypertriglyceridemia** | 0.55 [0.30, 1.00] | 0.051 | 0.69 [0.36, 1.33] | 0.269 |  |  |
| **Low HDL cholesterol** | 0.61 [0.40, 0.94] | **0.026** | 0.67 [0.42, 1.08] | 0.098 | 0.59 [0.38, 0.92] | **0.018** |
| **Unmet medical needs** | 0.37 [0.18, 0.75] | **0.007** | 0.35 [0.17, 0.73] | **0.006** | 0.66 [0.29, 1.51] | 0.315 |
| **Sustained depression for more than 2 weeks** | 0.99 [0.54, 1.85] | 0.984 | 1.16 [0.60, 2.30] | 0.669 |  |  |
| **Activity limitations** | 0.82 [0.47, 1.46] | 0.494 | 0.91 [0.48, 1.76] | 0.782 | 1.28 [0.66, 2.57] | 0.468 |
| **Interaction (Model 3)^d^** | **Unmet medical needs** | | **Activity limitations** | | 0.07 [0.00, 0.54] | **0.026** |

OR, odds ratio; CI, confidence interval; HDL, high-density lipoprotein

^a^Model 1 includes age, sex, and activity limitations as covariates, along with each variable in the column being added one at a time.

^b^Model 2 includes all the variables simultaneously to examine their combined effects on the outcome.

^c^Model 3 uses a two-step lasso: first selecting main effects, then considering interaction terms if warranted.

^d^In Model 3, activity limitation is not a significant variable on its own but may synergistically hinder adherence to PA guidelines when unmet medical needs are present. Without the interaction term, unmet medical needs become significant, whereas activity limitation remains non-significant.
