# Supplementary material for: Assessing adherence to physical activity guidelines and correlates among older Korean adults with a focus on 10-minute bout duration using subjective and objective measures
Source: PLoS One. 2025 Jun 9;20(6):e0324342. doi: 10.1371/journal.pone.0324342 (PMC12148133; doi:10.1371/journal.pone.0324342)
Supplement: Attachment — Submitted filename: PlosOne_review_PAguidelinesKorea.pdf [file pone.0324342.s004.pdf]

Assessing adherence to physical activity guidelines among older Korean adults with a focus on 10-minute bout duration using subjective and objective measures

This study aims to assess the adherence to PA guidelines in older Korean adults according to sociodemographic, lifestyle and health factors. PA was assessed by using a questionnaire and accelerometer recordings.

Review questions

Is the manuscript technically sound, and do the data support the conclusions?

Partly. Please see some comments.

Has the statistical analysis been performed appropriately and rigorously?

No. The statistical analyses are partly well performed. To complete the analyses, you could consider a model including interactions with a factor selection processing. Please consider the comments hereafter.

Have the authors made all data underlying the findings in their manuscript fully available?

Yes. It is written that this is available but the link provided leads to a blank page.

Is the manuscript presented in an intelligible fashion and written in standard English?

Yes. However, there are typos and some sentences should be revised.

Review Comments to the Author

General Comments

The study brings interesting information about the older Korean adult population, especially regarding PA habits through a study of factors associated with PA engagement.

However, some parts of the purpose of this study could be clearer (see details after). The results could be improved by using similar methods along the manuscript. Indeed, in the first part, there are comparisons between questionnaire, accelerometer > 1040 cpm and accelerometer > 1952 cpm whereas in the second part (Bland-Altman plots), the authors compared 1) questionnaire vs accelerometer > 1040 cpm and 2) accelerometer > 1952 cpm with 1 to 2 min tolerance. You could complete by adding information about "accelerometer > 1952 cpm with 1 to 2 min tolerance" in the first part and adding a Bland-Altman plot with "accelerometer > 1952 cpm".

More specifically, the statistical analyses could be refined (see details after).

The discussion could be improved. It is not clear what the authors could recommend about the method to use regarding the threshold. In addition, discussing the health factors associated with adherence could add value to the study.

Specific Comments

Abstract

Adherence to PA guidelines was evaluated using two different thresholds for the accelerometer data.

In the results, there are 3 thresholds (1952, 1040 and 560).

Financial disclosure

If possible, enter the initials of the authors who received the awards and the URL of the funders' website. Please, add the statement informing if the funders played any role in the study.

Data availability

The URL provided to access the data leads to a blank page (accessed on 22nd January 2025). Please, update the link.

Introduction

Line 88 – 89: I may miss something but I don't understand why you would like to get a more accurate alignment with questionnaire data as it was a gold-standard fir PA assessment. When reading the paper, it seems you compared accelerometer assessment with different parameters and you found that one is more aligned than the other one. Please rephrase.

Statistical analysis

Line 113: results related to 560 are not showed in the document.

Line 117 – 121: Why didn't you consider to use the "less stringent criterion" in the descriptive results and in the logistic models?

Line 162, 163: Please add a R citation in the text as recommended by the software providers.

Line 165: Make sure / check the use of t-tests is valid in each case. It may have been done since you used Welch t-tests in Table 2 (line 220).

Line 170: It seems you didn't consider the interaction factors in Model 2, especially age vs other factors, sex vs other factors, smoking vs hypertension, etc. You should add the significant ones in Models 2 and 3 (see a note hereafter) but the number of factors will increase (cf note below).

Line 172: "the primary analyses were replicated using different thresholds". Only one threshold is written. Please specify the thresholds you used.

Line 179: "accelrometer" -> "accelerometer" (typo).

Results

Line 186: "the study's participation process" (to be rephrased).

Line 208 and Table 2: Why didn't you consider the 560 cpm threshold? This threshold should probably be removed from the abstract and the methods.

Line 229 (Table 3), Line 257 (Table 4), Line 272 (Table 5): Model 2 involves many factors. The conditions for applying logistic regression may not be met since there only 133 participants with adherence (for Table 3). Suggestion: You might consider a Model 3 involving only factors with a p value less than a threshold ($p < 0.2$ for instance), such as "Drinking alcohol" (in Table 3). However, I don't think your conclusions will be changed.

Table 5 vs Fig. 2: The result message may be clearer if the criteria are similar, e.g. "1952 cpm, bouts lasting ≥ 10 min" or "1952 cpm, bouts lasting ≥ 10 min with 1 to 2 min tolerance".

Lines 285 – 287: You could help the reader to detect what brings the graph in your study.

Moreover, your manuscript includes only two graphs. What is the evidence showing that this threshold is optimal for minimizing the bias?

Discussion

Lines 325 – 332: What are your recommendations to readers? Should we consider that the associated factors are those highlighted with accelerometers, those highlighted with questionnaires, both? This is important to identify the factors on which public policy can focus on to address the lack of PA (cf Line 95).

Lines 339 – 341: Could you provide explanations or hypotheses regarding the association with these health factors?

Lines 342, 343: As highlighted in a comment about the introduction content, we could understand that using questionnaires is a gold-standard. Please rephrase.

Line 350 – 352: What evidence is there to support this assertion?

Line 350 – 358: What are your recommendations to readers? Which method is the best (threshold, with without tolerance)? How your results can support it?

Line 358: This may partly explain the discrepancies observed but did you get the information about the participant engagement in water-based physical activities?

Conclusion

Line 380, 381: What conditions enabled to get a minimal bias? This sentence is probably not appropriate since you did not compare biases according to factors but you compared the alignment according to algorithm parameters (thresholds) to detect MVPA. This should be rephrased.

Figures

Figure 2 and Figure 3: Please specify the units on the axes.

I find the shapes of the plots are a bit strange because of the linear pattern (like a triangle). Is there an explanation about these shapes?
