# Supplementary material for: Assessing adherence to physical activity guidelines and correlates among older Korean adults with a focus on 10-minute bout duration using subjective and objective measures
Source: PLoS One. 2025 Jun 9;20(6):e0324342. doi: 10.1371/journal.pone.0324342 (PMC12148133; doi:10.1371/journal.pone.0324342)
Supplement: Attachment — Submitted filename: Response to Reviewers.docx [file pone.0324342.s006.docx]

Response to the reviewers’ comments

**Manuscript ID:** PONE-D-24-02389

**Manuscript Title:** Assessing adherence to physical activity guidelines among older Korean adults with a focus on 10-minute bout duration using subjective and objective measures

We appreciate your thoughtful review of our manuscript, and have revised the manuscript according to the comments from you (Referees’ comments are shown in bold, and the newly added parts in the revised manuscript are marked in red).

Responses to the referees’ comments consist of three parts.

1. Editor (page 2 - page 4)

2. Reviewer #1 (page 5 - page 31)

3. Reviewer #2 (page 32 - page 57)

<Editor>

When submitting your revision, we need you to address these additional requirements.

1. Please ensure that your manuscript meets PLOS ONE's style requirements, including those for file naming. The PLOS ONE style templates can be found at

<https://journals.plos.org/plosone/s/file?id=wjVg/PLOSOne_formatting_sample_main_body.pdf> and [https://journals.plos.org/plosone/s/file?id=ba62/PLOSOne_formatting_sample_title_
authors_affiliations.pdf](https://journals.plos.org/plosone/s/file?id=ba62/PLOSOne_formatting_sample_title_authors_affiliations.pdf)

**Our Response:**

Thank you for the guidance. Following the editor’s suggestion, we revised the manuscript to fully comply with PLOS ONE’s style requirements, including the file naming conventions, and addressed all remaining points.

**2. Please note that PLOS ONE has specific guidelines on code sharing for submissions in which author-generated code underpins the findings in the manuscript. In these cases, all author-generated code must be made available without restrictions upon publication of the work. Please review our guidelines at** [**https://journals.plos.org/plosone/s/materials-and-software-sharing#loc-sharing-code**](https://journals.plos.org/plosone/s/materials-and-software-sharing#loc-sharing-code) **and ensure that your code is shared in a way that follows best practice and facilitates reproducibility and reuse.**

**Our Response:**

Thank you for your guidance regarding PLOS ONE’s code sharing policy. We would like to clarify that the SAS code used in our work is largely taken directly from the following publicly available resources, released under the Korean Open Government License Type 4 (KOGL 4), which requires attribution, prohibits commercial use, and does not allow modifications:

1. (Guidelines for the dataset “HN17_ALL”) Korea Disease Control and Prevention Agency. *Guidelines for using KNHANES VII 2017 raw data.* Korea Disease Control and Prevention Agency; 2018
2. (Guidelines for the dataset “HN17_PAM”) Korea Disease Control and Prevention Agency. *Guidelines for using raw data from the KNHANES accelerometer survey VII 2017.* Korea Disease Control and Prevention Agency; 2018

(Details for these two datasets can be found on the KNHANES website at <https://knhanes.kdca.go.kr/knhanes/eng/main.do> under Survey Data → Data Introduction. We apologize that we cannot provide a direct link that takes you to the exact page, as copying the link from that location does not open the site properly. We additionally ask for your understanding that, due to server instability, even the method we have confirmed to be working may eventually become unavailable.)

Regarding the SAS code, minor modifications were made to output processed versions of the HN17_ALL and HN17_PAM datasets for analysis, while the main analysis was performed using R. We contacted the Korea Disease Control and Prevention Agency (KDCA) by phone to resolve the apparent inconsistency in their guidelines (labeled under KOGL 4) and were informed that modifying and redistributing the publicly available SAS code is permitted. The restriction against modifying and redistributing applies to the guidance documents themselves, not to the SAS code.

In accordance with both the KOGL 4 and PLOS ONE’s requirements, we:

1. Acknowledged the source by citing the original code and license in our manuscript.
2. Shared the SAS code including our processed outputs, and include the license information so others are aware of the terms under which the code is provided (The SAS code used in our work is limited to initial data preparation steps for the KNHANES datasets).
3. Shared the R code without any access restrictions (The core analyses and primary findings were derived entirely using R).

We hope our code-sharing approach facilitates reproducibility, respects the original licensing terms, and satisfies PLOS ONE’s guidelines. Please let us know if you require any further information or clarification.

3. Thank you for stating the following financial disclosure:

“This work was supported by the National Research Foundation of Korea (NRF) grant funded by the Korea government (MSIT) (No. 2022R1A5A8023404).

This work was supported by the Pukyong National University Research Fund in 2022 (No. 202212470001).”

Please state what role the funders took in the study. If the funders had no role, please state: "The funders had no role in study design, data collection and analysis, decision to publish, or preparation of the manuscript."

If this statement is not correct you must amend it as needed.

Please include this amended Role of Funder statement in your cover letter; we will change the online submission form on your behalf.

**Our Response:**

Following the editor’s suggestion, we have revised the fund statement to include the requested Role of Funder Statement in the cover letter. Additionally, we have removed one of the previously listed funding sources as it is no longer applicable.

<Reviewer #1>

1. GENERAL REMARKS

**In the work entitled "Assessing adherence to physical activity guidelines among older Korean adults with a focus on 10-minute bout duration using subjective and objective measures" the authors sought to:**

**(i) analyze physical activity (PA) levels using both self-reported (via the validated K-GPAQ, Korea - Global Physical Activity Questionnaire) and objective accelerometer measures, as well as**

**(ii) investigate the factors associated with adherence to PA guidelines (using two different thresholds for the accelerometer data).**

**For this, the authors used data from the "Korea National Health and Nutrition Examination Survey (KNHANES VII-2) 2017" (aimed at individuals aged 65 years or older, who were capable of mobility and had consented to have their PA level measured by an accelerometer), in a study population consisting of n=363 older adults (average age 71.8 ± 4.74 yrs), across different socio-demographic characteristics. The authors conclude that: (i) discrepancies between self-reported and objective measurements were observed (more discrepant for the higher and lower cut-off values), and (ii) factors such as sex, age, and certain health conditions were significantly associated with PA adherence.**

**The study has its value. I will make some observations which can be of pertinence for the discussion regarding the study's more relevant inherent limitations and stronger points:**

**1) PA is not stable over longer periods which means that individuals identified as more or less active (or inactive) might not be consistently so; thus, it would be useful to perform longitudinal studies using objective measurements of PA, both at baseline and follow-up, with consistent measures over time;**

**2) The work** [**https://doi.org/10.1016/j.bbr.2020.113061**](https://doi.org/10.1016/j.bbr.2020.113061)**, by the same authors as REF 20, identified that >72% of studies evaluated PA "with self-report questionnaires, with limited validity in capturing the full range of daily living activities and with limitations associated with an inaccurate recall", while "in contrast, wearable sensors can provide an objective and precise assessment of everyday activities and may allow the identification of a specific PA pattern across time" in that "sensors incorporate measures of several activities including exercise, non-exercise, lifestyle activities (e.g., walking), and instrumental activities of daily life (e.g., housework). In general, these measures indicate the amount of total movement within an individual’s environment", concluding that "objective measures are extremely important to capture the low-intensity PA (particularly if considering that many older adults are sedentary)"**

**These aspects seem to be congruent with the authors observations (this reviewer does understand that the data used precludes the consideration of other measures across time), not taking away from the study, but enriching the body of literature.**

**Our Response:**

Thank you for your valuable comments. We admit that our cross-sectional design cannot account for fluctuations in PA over time. We also appreciate your reference to the benefits of wearable sensors, especially for detecting low-intensity activities in older adults. Following the reviewer’s suggestion,

1) we modified the Discussion section to highlight the importance of future work involving longitudinal studies with repeated objective measurements, which would enable capturing the variability of PA over time.

2) Additionally, we expanded on the limitations of self-reported methods—such as recall bias and underestimation of low-intensity activities—in the Introduction of the revised manuscript.

The modifications on the revised manuscript are as follows:

- Lines 483-487: *Lastly, as PA levels are not necessarily stable over longer periods, individuals classified as more or less active at a single time point may not consistently remain so over time. Consequently, the cross-sectional nature of this study limits our ability to capture changes in PA behavior. Future research should consider longitudinal designs with objective PA measurements at multiple time points, ensuring consistent measures over time to more accurately capture temporal variations in activity levels.*
- Lines 52-54: *However, these subjective tools have inherent limitations in accurately capturing the full range of daily living activities and are prone to recall bias [15].*
- Lines 55-57: *They can measure various type of activities, and even capture low-intensity activities objectively and accurately [15,18].*

**2. ABSTRACT AND INTRODUCTION**

**Well written and aligned with the work. Given the existing body of literature, I would suggest that the introduction would benefit from including references to a couple of systematic reviews and/or meta-analysis on the matter, rather than limiting to individual studies (regardless of their merit). My suggestion derives from the fact that literature and guidelines seem to be rather US-centric (see your suggested REFS 5-8), and perhaps larger studies could provided for more generalizable/over-reaching conclusions. I realize that if those are based on US-centric studies/guidelines that there is also an argument here against it. I will, thus, leave this up to the authors.**

**Our Response:**

Thank you for your comment. Although REFS 5-8 in the previous manuscript include guideline from both the UK and Canda, we acknowledge the importance of incorportating broader perspectives. Following the reviewer’s suggetion, we have added references to a systematic comparative analysis of PA guidelines in EU countries [REF 9], as well as those relevant to Koreans [REF 10], to provide a more comprehensive and globally representative context. The modifications on the revised manuscript are as follows:

- Lines 43-48: *Therefore, increasing PA levels has been recommended as public guidelines in many organizations and countries [5–10]. For instance, the World Health Organization [8] and the Korean Ministry of Health and Welfare [10] recommend that, for substantial health benefits, all adults aged 18 or over should perform at least 150 to 300 minutes of moderate-intensity aerobic PA or at least 75 to 150 minutes of vigorous-intensity aerobic PA, or an equivalent combination of moderate- and vigorous-intensity activity throughout the week.*

Additional references:

[9] Gelius P, Tcymbal A, Abu-Omar K, Mendes R, Tribuzi Morais S, Whiting S, et al. Status and contents of physical activity recommendations in European Union countries: a systematic comparative analysis. BMJ Open. 2020;10: e034045.

[10] Ministry of Health and Welfare, Korea Health Promotion Institute. Physical activity guideline for Koreans, revised edition. 2023. Available: <https://www.mohw.go.kr/board.es?mid=a10411010100&bid=0019&act=view&list_no=1479208&tag=&nPage=1>

**3. I do, however, wonder: are there recommendations in Korea by, for instance, a health ministry (or equivalent government/health authority)? My question is not such a rethorical one - the paper proposes to assess "adherence to physical activity guidelines" - thus, it begs the question: whose (which) guidelines?**

**Our Response:**

Thank you for your valuable comment. We have clarified that physical activity guidelines for Koreans align with the WHO recommendations. To reflect this, we have revised the sentence accordingly as follows:

- Lines 44-48: *For instance, the World Health Organization [8] and the Korean Ministry of Health and Welfare [10] recommend that, for substantial health benefits, all adults aged 18 or over should perform at least 150 to 300 minutes of moderate-intensity aerobic PA or at least 75 to 150 minutes of vigorous-intensity aerobic PA, or an equivalent combination of moderate- and vigorous-intensity activity throughout the week.*

**4. Of note, this objective is re-stated somewhat differently in lines 87 and 88, in which it is stated that the study aim is to "explore PA levels among older adults in Korea, using both self-reported questionnaires and accelerometer measurements".**

**Our Response:**

Thank you for your important comment. We have clarified and restated the purpose of our study by distinguishing between the primary and secondary objectives. The primary objective of this study to explore PA adherence among older adults in Korea using both self-reported questionnaires and accelerometer measurements. The secondary objective is to explore factors associated with PA adherence based on both measurement tools within this context. The modifications on the revised manuscript are as follows:

- Lines 93-95: *The primary objective of this study is to explore PA adherence among older adults in Korea, using both self-reported questionnaires and accelerometer measurements. The secondary objective is to investigate factors associated with this PA adherence based on both measurement tools.*

**5. I would make sure to align title, with objectives stated in the abstract, with objectives stated in the concluding paragraph of the introduction, and clearly state the guidelines using to determine/assess Adherence/Nonadherence.**

**Our Response:**

Thank you for your important comment. We have clarified and restated the purpose of the study by distinguishing between the primary and secondary objectives, ensuring alignments with the title. The primary objective of this study to explore PA adherence among older adults in Korea using both self-reported questionnaires and accelerometer measurements. The secondary objective is to explore factors associated with PA adherence based on both measurement tools. Following the reviewer’s suggestion, we have revised the manuscript as follows:

- Title: *Assessing adherence to physical activity guidelines and correlates among older Korean adults with a focus on 10-minute bout duration using subjective and objective measures*
- Lines 19-22 (Abstract): *The primary objective of this study was to analyze PA levels in older Korean adults using both self-reported and objective accelerometer measures, and the secondary objective was to investigate the factors associated with adherence to PA guidelines.*
- Lines 93-95: *The primary objective of this study is to explore PA adherence among older adults in Korea, using both self-reported questionnaires and accelerometer measurements. The secondary objective is to investigate factors associated with this PA adherence based on both measurement tools.*
- Lines 159-174 (a new subsection named Physical activity adherence has been added): *PA adherence was defined as engaging in 150 to 300 minutes of moderate-intensity aerobic PA or at least 75 to 150 minutes of vigorous-intensity aerobic PA, or an equivalent combination of moderate- and vigorous-intensity activity throughout the week, as measured using K-GPAQ. For the accelerometer-based assessment, we analyzed four distinct scenarios to capture MVPA bouts of at least 10 consecutive minutes, differentiated by cutoff values (1952 cpm or 1040 cpm) and the allowance of a brief tolerance period (1–2 minutes) within each bout. Specifically, the four scenarios are:*

*a) 1952 cpm 10-minute bout: Participants must maintain ≥1952 counts per minute for the full 10-minute period.*

*b) 1952 cpm 10-minute bout with a 1–2-minute tolerance: Participants must maintain ≥1952 cpm for at least 8 out of 10 minutes, permitting up to 2 minutes below the threshold.*

*c) 1040 cpm 10-minute bout: Participants must maintain ≥1040 cpm for the entire 10-minute interval.*

*d) 1040 cpm 10-minute bout with a 1–2-minute tolerance: Participants must maintain ≥1040 cpm for at least 8 out of 10 minutes, allowing up to 2 minutes below the threshold.*

*If the total MVPA minutes computed from the accelerometer reached or exceeded 150 minutes per week under any scenario, the participant was considered to have adhered to the PA guidelines.*

**6. MATERIALS AND METHODS**

**In line 99, a "which" is required after the comma so to read ", which was accessed for research purposes on February 15th 2023". (note: I will not further dwell on minor grammatical lapses, as certainly a careful reading by the authors will identify those in latter phases);**

**Our Response:**

Thank you for your comment. During the revision of the manuscript—aimed at providing a more detailed description of the dataset (see Comment #7 below)—the sentence was substantially modified, and the revised version is as follows:

- Lines 123-125: *Ultimately, we acquired a subset of PA monitoring data comprising 425 participants (197 men, 189 women) who had at least three valid days of accelerometer data from the KNHANES VII-2 2017 dataset, which was accessed for research purposes on February 15th 2023.*

**7. Are there other studies using the KNHANES VII-2 2017 dataset? Can references be provided? The KNHANES VII-2 2017 was coordinated by whom? It covered what percentage of the population? Was it country wide? Some general descriptives of the KNHANES VII-2 2017 are needed. I am also confused on this supplemented other self-reported PA? Was it KNHANES VII-2 2017 run after another survey, adding to the data? Lines 98 to 104 are essential in understanding the dataset at hand;**

**Our Response:**

Thank you for your valuable comment. We recognized that a more detailed description of the dataset is necessary. KNHANES has been the representative national database in Korea since 1998 and numerous studies have utilized KNHANES VII data for various research purposes. Given the extensive number of studies using this data, we have not cited specific references in the main text; rather, we have highlighted its broad applicability for research purposes (https://knhanes.kdca.go.kr/knhanes/eng/thss/thssInfLst.do).

Examples of research using KNHANES VII-2 2017 dataset :

Kim Y, Kim DW, Kim K, Choe JS, Lee HJ. Usual intake of dietary isoflavone and its major food sources in Koreans: Korea National Health and Nutrition Examination Survey 2016-2018 data. Nutr Res Pract. 2022 May;16(Suppl 1):S134-S146.

Kim EK, Cho JY, Park EY. Association between periodontal disease and hypertriglyceridemia: Propensity score matching analysis using the 7th Korea National Health and Nutrition Examination Survey. Medicine (Baltimore). 2023 Dec 22;102(51):e36502.

Jeong S, Lee H, Jung S, Kim JY, Park S. Higher energy consumption in the evening is associated with increased odds of obesity and metabolic syndrome: findings from the 2016-2018 Korea National Health and Nutrition Examination Survey (7th KNHANES). Epidemiol Health. 2023;45:e2023087.

Gwak DY, Lee SA. Lifestyle Behaviors According to the Duration of Hypertension: Korea National Health and Nutrition Examination Survey 2016-2018. J Korean Med Sci. 2022 Dec 19;37(49):e343. doi: 10.3346/jkms.2022.37.e343. PMID: 36536544; PMCID: PMC9763709.

Seo BY, Her ES. Regional disparities related to cardiovascular diseases and diet quality in Korean adults: based on the 2013-2016 Korea National Health and Nutrition Examination Survey Data. Nutr Res Pract. 2022 Dec;16(6):755-764.

Chang K, Yoo S, Lee S. Classification and prediction of the effects of nutritional intake on diabetes mellitus using artificial neural network sensitivity analysis: 7th Korea National Health and Nutrition Examination Survey. Nutr Res Pract. 2023 Dec;17(6):1255-1266

Full dataset of KNHANES represents the national population through a complex, stratified probability sample, comprising 23 households from 192 regions, and approximately 8127 household members aged one year and older, accounting for about 0.015 % of the total population in South Korea. However, for analysis, we used only a subset of 491 older adults (≥ 65 years) who consented to accelerometer-based PA assessment. We integrated sociodemographic and health-related information from the full KNHANES dataset (HN17_ALL) with the the accelerometer-measurment PA (HN17_PAM) subset based on the participatns’ ID, allowing for a combined analysis of subjective and objective PA measurements.

Consequently, because our analysis was limited to this subset of older adults who consented to accelerometer-based PA assessment, we did not include a detailed description of the original sampling methods and population coverage in the Materials and Methods section. Following the reviewer’s suggestion, we have clarified the dataset description (KNHANES), including its coordinating agency, survey content, and the merging of datasets for analysis. In addition, to better explain the study's workflow, we relocated the flow chart (Fig 1. Flow chart utilized to identify eligible male and female participants for the study) from the Results section to this part. The modifications on the revised manuscript are as follows:

- Lines 98-127: *The Korea National Health and Nutrition Examination Survey (KNHANES) is an annual nationwide health survey conduced since 1998 by the Korea Disease Control and Prevention Agency. It aims to produce represenatative and reliable statistics on the health and nutritional status of the Korean population. This survey is publicily available annually and has been widely used in research to assess the epidemiology of chronic diseases, analyze health and nutritional status, examine socioeconomic disparities, evaluate the effectiveness of health intervention in policy making.*

*The first dataset, HN17_ALL [35], collects data through physical examinations, measurements, and laboratory tests, as well as through health interviews covering sociodemographic, healh-behavioral, and nutritional information, including self-reported PA levels measured using the Korean GPAQ (K-GPAQ) [13]. In the second dataset, HN17_PAM [36], objective PA levels were measured via accelerometers in a subset of 491 older adults (≥ 65 years) who were physically capable of mobility and consented to accelemeter-based PA measurement. This accelerometer dataset was provided as part of the KHNAES VII-2 2017. For our analysis, we integrated sociodemographic and health-related information from the full KNHANES dataset (HN17_ALL) with the accelerometer-based PA (HN17_PAM) subset using participatns’ IDs, allowing for a combined analysis of subjective and objective PA measurements.*

*Fig 1 presents the selection process used to derive our analytic sample from the whole dataset, illustrating how older adults (≥65 years) meeting the study’s inclusion criteria were identified and further filtered based on accelerometer-based PA data availability. From the dataset (N = 8,127), a total of 6,456 individuals under 65 years of age were excluded, leaving 1,671 older adults (≥ 65 years). Of these, 1,180 did not consent to accelerometer-based PA measurement, resulting in 491 participants who were initially invited to wear an accelerometer. Among those invited, 17 were excluded due to device loss or malfunction, noncompliance, or data errors, and another 49 were excluded for having fewer than three valid days of accelerometer wear time. For participants who did not have 7 days of valid wear, the total minutes of MVPA were adjusted according to the number of valid days they had. Ultimately, we acquired a subset of PA monitoring data comprising 425 participants (197 men, 189 women) who had at least three valid days of accelerometer data from the KNHANES VII-2 2017 dataset, which was accessed for research purposes on February 15th 2023.*

***Fig 1. Flow chart utilized to identify eligible male and female participants for the study.*** KNHANES: Korea National Health and Nutritional Examination Survey, PA: physical activity

- Figure 1:

Additional references:

[35] Korea Disease Control and Prevention Agency. Guidelines for using KNHANES VII 2017 raw data. 2018. Available: <https://knhanes.kdca.go.kr/knhanes/eng/main.do>

[36] Korea Disease Control and Prevention Agency. Guidelines for using raw data from the KNHANES accelerometer survey VII 2017. 2018. Available: <https://knhanes.kdca.go.kr/knhanes/eng/main.do>

**8. Please better explain what is considered to be "capable of mobility" - there are different levels of mobility. This variable in itself may be of great consideration - in line 169 when you explain your models, I am tempted to argue that Model 1 should account for heterogeneity in mobility, and only then run other Models (including age and biological sex);**

**Our Response:**

Thank you for your insightful comment. By incorporating the spirit of your suggestions as thoroughly as possible, we believe the overall quality of our paper has been significantly enhanced. In the following, we explain this improvement process in detail.

First of all, the *Guidelines for using raw data from the KNHANES accelerometer survey VII 2017* (i.e., guidelines for the accelerometer dataset HN17_PAM) [36] only specify that individuals who are incapable of mobility were excluded from the survey. It does not provide explicit criteria regarding different levels of mobility.

At the same time, the full KNHANES dataset (HN17_ALL) [35], which includes questionnaire data (K-GPAQ), also features a question related to mobility. The question translates as follows:

“Do you have any limitations on daily life and social activities due to health problems, physical disabilities, or mental disabilities?”

Since the guideline for HN17_PAM does not provide a direct measure of mobility levels, we can use this self-reported question on activity limitations as a proxy for mobility status. While this does not capture specific gradations of mobility such as walking speed or balance issues, it provides an indication of whether participants experience functional mobility constraints in daily activities. Therefore, we may operationally define ‘capable of mobility’ as not having self-reported activity limitations in this study.

In fact, we previously conducted our analysis only on participants who answered that they had no such restrictions. As shown in Figure 1 in the previous manuscript, among the 425 participants with at least three valid wear days, we selected 363 individuals without activity restrictions (term now standardized to “activity limitation” for consistency) to analyze both questionnaire and accelerometer data. This was based on the assumption that individuals with daily activity limitations would naturally have lower PA levels, making it unnecessary to examine this through our study. Previously, we focused on the so-called “active senior” population and investigated other factors influencing adherence to PA guidelines.

Following the reviewer’s suggestion, we included the activity limitation covariate in all our models to account for mobility heterogeneity in the entire analysis. Surprisingly, our initial expectations were not met. Although activity limitation proved to be a significant variable in the questionnaire data (Table 3), it did not reach significance in the accelerometer data (Tables 4-5). Considering that the activity limitation item, like the K-GPAQ, is based on subjective responses, these findings imply that the questionnaire data may not solely reflect actual PA but also incorporate respondents’ subjective perceptions.

The subjectivity of questionnaire data can affect covariate analysis. More specifically, certain covariates—such as education—may contribute to differences in self-reported adherence to PA guidelines, potentially reflecting variations in reporting tendencies rather than actual adherence. In contrast, accelerometer data provide an objective measure, eliminating the potential confounding effects regarding self-reported tendencies. This highlights a key advantage of accelerometer data in identifying factors influencing PA adherence. In summary, our study’s findings capture the inherent subjectivity of questionnaire data. Therefore, in the context of identifying covariates that influence adherence to PA guidelines, we conclude that accelerometer data are more appropriate. Regarding this point, we provide further elaboration in Comment 15 below.

In response to the reviewer’s comment, we included activity limitation as an additional covariate in all models and revised the manuscript as follows:

- Lines 177-181: *Sociodemographic variables included age, sex, education, household income, economic activity, and marital status, and health-related variables included smoking, alcohol consumption, body mass index (BMI), hypertension, diabetes, hypercholesterolemia, hypertriglyceridemia, and low* *high-density lipoprotein (HDL) cholesterol, unmet medical needs, depression, and activity limitation.*
- Lines 200-202: *Activity limitation was defined as having any limitation in daily life or social activities due to health problems, physical disabilities, or mental disabilities.*
- Lines 213-215: *Model 1 included age sex, and activity limitations as baseline covariates and then assessed the effect of each additional covariate individually, thereby representing a minimal evaluation of each variable’s independent association.*
- Lines 293-305: *In Model 1, higher education (OR=1.36 [1.12, 1.65]) and standardized household income (OR=1.33 [1.08, 1.65]) were each linked to higher odds of PA adherence, while older age (OR=0.95 [0.91, 1.00]) and activity limitations (OR=0.41 [0.19, 0.78]) emerged as negative correlates. In Model 2, the following variables were positively linked to meeting the PA guideline: being male (OR=2.37 [1.09, 5.24]), having higher educational attainment (OR=1.28 [1.03, 1.58]), and being diagnosed with hypercholesterolemia (OR=1.70 [1.05, 2.75]). Conversely, participants with activity limitations were significantly less likely to meet the guideline (OR=0.45 [0.20, 0.92]). Finally, Model 3, which was derived using a two‐stage LASSO selection procedure, highlighted the following set of variables—education (OR=1.32 [1.08, 1.60]), standardized household income (OR=1.26 [1.02, 1.57]), and hypercholesterolemia (OR=1.73 [1.10, 2.71]) as positive correlates; hypertension (OR=0.60 [0.39, 0.93]) and activity limitations (OR=0.45 [0.21, 0.89]) as negative correlates. Although there were slight differences in the significant variables among the three models, they exhibited an overall similar pattern.*
- Lines 454-462: *In the logistic regression analysis, we explored a potential interaction using LASSO in Model 3. For the most part, the main findings did not reveal any significant interactions (Tables 3–5). However, when applying accelerometer data with a >1040 cpm cutoff, ≥10-minute bouts, and a 1–2-minute tolerance (Table S2), we detected significant interactions involving unmet medical needs and activity limitation. Specifically, although activity limitation on its own was not significant, it exerted a synergistic effect when combined with unmet medical needs, resulting in a substantial decrease in PA adherence. In general, it is relatively uncommon to observe an interaction effect. Moreover, given the large number of potential interaction terms tested, this finding may have arisen by chance (i.e., multiple testing). Hence, it should be interpreted with caution, and further research is required to confirm it.*
- Lines 412-414: *This subjectivity can be further supported by the observation that the activity limitation covariate, which is measured by questionnaire, also emerges as significant in the questionnaire data (Table 3) but not in the accelerometer data (Tables 4–5).*
- All Tables (1~5): Results for activity limitations are added.
- Fig 1: Due to the removal of the step that excluded participants reporting activity limitation on the survey, the final flowchart was updated to analyze data from 425 participants instead of 363.

**9. Please provide the reasoning for the cpm values (>560, >1040, >1952) in conjunction with a 10-minute bout criterion. Did you follow other studies (for instance, REF 16 that you discuss in your intro)? Did you follow some pre data analysis that indicated for this cut-offs/tresholds? That is: where do these specific numbers came from?**

**Our Response:**

Thank you for your comment. The >1952 cpm cut point was referenced from studies on accelerometer-based physical activity data in middle-aged adults [REFs 31–33]. The >1040 cpm cut point was referenced from studies on accelerometer-based PA data in older adults [REF 27]. In contrast, the >560 cpm threshold was arbitrarily set for exploratory purposes. We agree with the reviewer’s recommendation to use only the criteria provided in the references and to disclose them transparently.

In response to the reviewer’s comment, we have decided to include only the reference-based cut points documented in the literature [27, 31–33] and to incorporate a 1–2-minute tolerance when defining a 10-minute bout [34]. We have also provided a more detailed explanation of these criteria while citing the references more directly. As a result, when addressing the accelerometer data, we focus on four clear scenarios:

a) 1952 cpm 10-minute bout

b) 1952 cpm 10-minute bout with a 1–2-minute tolerance

c) 1040 cpm 10-minute bout

d) 1040 cpm 10-minute bout with a 1–2-minute tolerance.

Moreover, we previously structured the manuscript to recommend a specific combination of cutoff and tolerance criteria for accelerometer data based on the adherence rates reported in the questionnaire data. However, during the revision process, as we became more aware of the potential issues arising from the subjectivity of the questionnaire data, this approach was abandoned. Therefore, we do not endorse any single combination (a~d) as being more favorable for Korean older adults.

Following the reviewer's suggestion, the following modifications have been made to the revised manuscript:

- Lines 82-92: *In this study, we incorporated both the commonly used cutoff level of >1952 cpm for MVPA in middle-aged adults [31–33] and the >1040 cpm threshold derived from a laboratory-based calibration study in an older adult population [27]. In the latter study, the cutoff was established based on oxygen consumption—a representative health indicator of PA—and no significant sex differences were observed in either activity counts or oxygen consumption, thereby eliminating the need for sex-specific criteria. Meanwhile, because the GPAQ generally measures aerobic PA in bouts of at least 10 minutes, PA adherence—defined as achieving ≥150 minutes of MVPA within 7 days—was assessed by summing the total duration of continuous MVPA bouts of 10 minutes or more. In addition, we evaluated an alternative approach that allows a 1–2-minute tolerance when defining 10-minute bouts [34], leading to four scenarios: two cutoffs (1952 cpm and 1040 cpm), each applied with and without the 1–2-minute tolerance.*
- Lines 162-174: *For the accelerometer-based assessment, we analyzed four distinct scenarios to capture MVPA bouts of at least 10 consecutive minutes, differentiated by cutoff values (1952 cpm or 1040 cpm) and the allowance of a brief tolerance period (1–2 minutes) within each bout. Specifically, the four scenarios are:*

*a) 1952 cpm 10-minute bout: Participants must maintain ≥1952 counts per minute for the full 10-minute period.*

*b) 1952 cpm 10-minute bout with a 1–2-minute tolerance: Participants must maintain ≥1952 cpm for at least 8 out of 10 minutes, permitting up to 2 minutes below the threshold.*

*c) 1040 cpm 10-minute bout: Participants must maintain ≥1040 cpm for the entire 10-minute interval.*

*d) 1040 cpm 10-minute bout with a 1–2-minute tolerance: Participants must maintain ≥1040 cpm for at least 8 out of 10 minutes, allowing up to 2 minutes below the threshold.*

*If the total MVPA minutes computed from the accelerometer reached or exceeded 150 minutes per week under any scenario, the participant was considered to have adhered to the PA guidelines*

**10. Did you use the GPAQ or the K-GPAQ? (please, compare and contrast information on line 123 and 130;**

**Our Response:**

Thank you for your comment here. We used the K-GPAQ, and all parts referring to its use have been revised accordingly. The modifications on the revised manuscript are as follows:

- Line 130: *Questionnaire data were collected from participants using the K-GPAQ.*
- Line 231:

$$Mean=\frac{K-GPAQ minutes of MPVA per day + accelerometer minutes of MVPA per day}{2}$$

- Line 232:

$$Difference=K-GPAQ minutes of MVPA per day - \mathrm{accel}erometer minutes of MVPA per day.$$

- Table 2: When describing the questionnaire data, we have designated them as K-GPAQ.
- Lines 361-363: ***Fig 2. Bland-Altman plots for daily MVPA minutes comparing the questionnaire data (Q = K-GPAQ) with accelerometer data under the following settings: A. A1952 (>1952cpm). B. A1952t (>1952cpm, tolerated). C. A1040 (>1040cpm). D. A1040t (>1040cpm, tolerated).***

**11. Why the variables on lines 139 to 143? Why were these specific ones selected from the KNHANES VII-2 2017 information source? Again, was it based on literature? Did you run some statistical analysis and these were the most significant? It is ok to show data for the most significant, but it would be great to also understand if there were others that were run but without significant results (in itself a finding), or the reasoning behind "picking" some and not others (albeit I do not think that being "significant" was your reasoning). The statement in line 159 a 160 does not withstand the test of scientific rigor on this (please provide REFS or a more sustainable/sound argument);**

**Our Response:**

Thank you for the comment. We understand the concerns regarding selecting variables from the KNHANES VII-2 2017 dataset. In general, variables can be chosen based on existing literature, statistical significance, or a purely data-driven approach. In order to provide a more detailed explanation, we respectfully note that in finalizing which variables were deemed most pertinent to our research topic, some element of researcher judgment was unavoidably involved for the reasons outlined below.

Initially, we faced the challenge of having over 800 potential covariates in the raw dataset (HN17_ALL), whereas our sample size was limited to 363 observations (later 425 in the revised manuscript). Specifically, the dataset includes numerous items—ranging from those that appear less obviously related (e.g., bicycle helmet use, seat belt use while driving) to many others that are more likely to correlate with our variables of interest (e.g., drunk driving). There were also many sub-items regarding the survey questions. A fully data-driven approach that attempts to incorporate all 800 potential covariates in the variable selection process would be practically infeasible, since the number of parameters would far exceed the number of observations. Moreover, without properly refining these numerous variables, the model may be prone to overfitting or spurious patterns, even leaving aside the problems of missing values and multicollinearity in the data.

To address these limitations, we conducted preliminary screenings that involved both (1) domain knowledge, drawing on prior studies suggesting certain health behaviors and socioeconomic indicators might be relevant (e.g., Kortajarena et al. 2019 [REF 22], Jefferis et al. 2014 [REF 33]), and (2) statistical checks (e.g., variance inflation factor, missingness rates) to narrow down the list to 16 plausible covariates. This pre-selection reduced the dimension of the problem to a level compatible with our sample size. We admit that this approach seems less data-driven. That said, given the extensive range of variables available and the constraints of our dataset, applying domain expertise to narrow down the pool of potential covariates was necessary for a robust and interpretable result.

Since our secondary objective is to explore whether the variable correlates with adherence, we chose not to restrict ourselves solely to variables already identified as significant in the literature, as doing so could limit the scope of our research. We selected medical conditions that we wished to investigate for their potential relationship with PA as our variables of interest. In other words, among the various conditions available, we focused on those that appeared most plausibly related to PA. This is not intended to assert causality; rather, it proposes potential candidates that may inspire future research. Regarding the variables that were excluded, most of the excluded variables were ones that would likely not be accepted under common standards. That is, even if a correlation with the outcome variable were observed, it would generally be interpreted as either an incidental finding due to chance or an inexplicable confounding effect of other variables, thus providing relatively little impetus for further investigation. In conclusion, we sincerely request your understanding that, given the inherent nature of our research topic and dataset, it is not feasible to implement a flawless approach in terms of scientific rigor—one governed solely by consistent rules or data—and that certain decisions necessarily relied on the researcher’s discretion.

To address the reviewer’s concern, we implemented an additional analytical step using a LASSO-based approach on the 17 selected variables and their potential interaction terms to further refine the model (as mentioned in Comment #8, we have added the “activity limitation” covariate, and as a result, the revised manuscript now covers a total of 17 variables). LASSO (least absolute shrinkage and selection operator) is a method that applies an L1 penalty to the regression coefficients, driving some of them to zero and thereby achieving both variable selection and model regularization simultaneously. This technique effectively reduces the number of variables based on the data. Although LASSO can be applied in high-dimensional settings (where the number of variables overwhelmingly exceeds the number of data points), in our research context, challenges may arise as sensitivity to such settings can lead to unstable results. Specifically, small changes in the optimization settings can result in drastic variations in the combination of selected variables, making it difficult to generalize.

As a result, we introduced “Model 3,” which considered all 17 chosen covariates plus their interactions, and then applied LASSO to select only the statistically significant predictors. The model was constructed by first performing a LASSO logistic regression with only first-order terms, selecting 3–5 variables based on the LASSO path. Then, all variables and their their interaction terms were entered into another LASSO analysis, allowing for the inclusion of one possible additional variable. The final model comprises the initially selected variables plus any significant interaction terms (and their main effects if not already included). This procedure not only helps identify significant variables and detect potential interactions, but also provides results that are relatively stable and generalizable.

We hope this clarifies the rationale for our variable selection method and addresses your concerns. If you require further details or additional approaches, we would be happy to provide them. The modifications on the revised manuscript are as follows:

- Lines 181-183: *These variables were carefully selected from among the extensive KNHANES dataset based on previous research (e.g., [22,33]) and preliminary statistical assessments to ensure both generalizability and analytical feasibility.*
- Lines 217-223: *Model 3 employed a two-step LASSO logistic regression approach: first, only first-order terms were considered, selecting three to five variables based on the LASSO path; second,* *all variables along with their interaction terms were entered into another LASSO analysis, allowing for the inclusion of one possible additional variable. The final model thus comprised those initially selected variables plus any significant interaction terms (and their main effects if not already included). This multi-model strategy enabled us to explore both minimal and comprehensive covariate adjustments, as well as to systematically identify potentially important interactions in a data-driven manner.*
- Lines 300-304: *Finally, Model 3, which was derived using a two‐stage LASSO selection procedure, highlighted the following set of variables—education (OR=1.32 [1.08, 1.60]), standardized household income (OR=1.26 [1.02, 1.57]), and hypercholesterolemia (OR=1.73 [1.10, 2.71]) as positive correlates; hypertension (OR=0.60 [0.39, 0.93]) and activity limitations (OR=0.45 [0.21, 0.89]) as negative correlates.*
- Lines 313-319: *Finally, Model 3 yielded a more parsimonious selection of significant predictors, retaining sex (OR=2.23 [1.40, 3.57]) while identifying two lipid‐related factors—hypertriglyceridemia (OR=0.30 [0.10, 0.74]) and low HDL (OR=0.33 [0.19, 0.56])—as significant negative correlates. Although unmet medical needs and economic activity were selected during the LASSO variable selection process, they did not reach statistical significance in the final model constructed with the chosen variables. It is worthwhile noting that a non-significant result does not imply the absence of an effect; the corresponding OR values can remain meaningful.*
- Lines 335-336: *Finally, in Model 3, male (OR=2.19 [1.44, 3.34]), diabetes (OR=0.60, [0.36, 0.97]), and low HDL (OR=0.41 [0.26, 0.64]) emerged as significant predictors.*
- Lines 454-462: *In the logistic regression analysis, we explored a potential interaction using LASSO in Model 3. For the most part, the main findings did not reveal any significant interactions (Tables 3–5). However, when applying accelerometer data with a >1040 cpm cutoff, ≥10-minute bouts, and a 1–2-minute tolerance (Table S2), we detected significant interactions involving unmet medical needs and activity limitation. Specifically, although activity limitation on its own was not significant, it exerted a synergistic effect when combined with unmet medical needs, resulting in a substantial decrease in PA adherence. In general, it is relatively uncommon to observe an interaction effect. Moreover, given the large number of potential interaction terms tested, this finding may have arisen by chance (i.e., multiple testing). Hence, it should be interpreted with caution, and further research is required to confirm it.*
- Tables 3-5: Model 3 has been added to each table.

**12. RESULTS**

**- On "Table 2" for the nomenclature/line name for the parameter "Physical activity adherence - Questionnaire" can be conducive to misinterpretation. For instance, this reviewer read the table in that not all participants have all measures (in this case that on=230 did not have data), while it means that 63.4% (n = 230) did not meet the guidelines (as explained in the paragraph). I would rename the line, or add a footnote, so that it is clearer.**

**Our Response:**

We apprecitate your important comment. We have revised Table 2 by renaming the line to “PA Adherence” “Met vs Not met” and adding a footnote to clarify the interpretation as follows (Line 250).

In addition, regarding Table 2, we found that the lower section of Table 1 (PA adherence) in the original manuscript and the upper section of Table 2 contained identical content and test results. Therefore, to avoid redundancy, we removed the lower section of Table 1.

**13. CONCLUSION/DISCUSSION**

**- In the opening paragraph, the authors have caused some confusion on this reviewer. I was operating under the assumption (and the manuscript led me to it) that the authors used a "subset" of data of the larger survey KNHANES VII-2 2017. In fact, in the results lines 186 to 192, and Figure 1, I continued to read under this assumption, stemming from the materials & methods. However, in lines 307 to 308, now it clearly states that** **the study authors recruited the participants. I must stress the need to please better describe these samples: KNHANES VII-2 2017 vs. study sample - it has to be very clear.**

**Our Response:**

We sincerely apologize for any confusion caused by our previous explanation, and we appreciate this opportunity to address your important comments. As mentioned in Comment #7, the KNHANES VII-2 dataset is divided into several parts. In our study, we used a full dataset that excluded accelerometer data (HN17_ALL) and a subset containing the accelerometer data from participants who consented (HN17_PAM). We utilized the provided dataset and did not independently recruit participants. We acknowledge that our description of the study sample—particularly in lines 307 to 308 of the original manuscript where it was stated that the study authors recruited the participants—may have led to misunderstandings.

To rectify this, we have revised the Materials and Method section to provide a clearer description of KNHANES VII-2 (2017). Additionally, we have updated Fig 1 (flow chart) and relocated it from the Results section to the Materials and Methods section to better illustrate the process. Finally, we have revised the statement in lines 307-308 of the original manuscript. The modifications in the revised manuscript are as follows:

- Lines 98-127: *The* *Korea National Health and Nutrition Examination Survey (KNHANES) is an annual nationwide health survey conduced since 1998 by the Korea Disease Control and Prevention Agency. It aims to produce represenatative and reliable statistics on the health and nutritional status of the Korean population. This survey is publicily available annually and has been widely used in research to assess the epidemiology of chronic diseases, analyze health and nutritional status, examine socioeconomic disparities, evaluate the effectiveness of health intervention in policy making.*

*The first dataset, HN17_ALL [35], collects data through physical examinations, measurements, and laboratory tests, as well as through health interviews covering sociodemographic, healh-behavioral, and nutritional information, including self-reported PA levels measured using the Korean GPAQ (K-GPAQ) [13]. In the second dataset, HN17_PAM [36], objective PA levels were measured via accelerometers in a subset of 491 older adults (≥ 65 years) who were physically capable of mobility and consented to accelemeter-based PA measurement. This accelerometer dataset was provided as part of the KHNAES VII-2 2017. For our analysis, we integrated sociodemographic and health-related information from the full KNHANES dataset (HN17_ALL) with the accelerometer-based PA (HN17_PAM) subset using participatns’ IDs, allowing for a combined analysis of subjective and objective PA measurements.*

*Fig 1 presents the selection process used to derive our analytic sample from the whole dataset, illustrating how older adults (≥65 years) meeting the study’s inclusion criteria were identified and further filtered based on accelerometer-based PA data availability. From the dataset (N = 8,127), a total of 6,456 individuals under 65 years of age were excluded, leaving 1,671 older adults (≥ 65 years). Of these, 1,180 did not consent to accelerometer-based PA measurement, resulting in 491 participants who were initially invited to wear an accelerometer. Among those invited, 17 were excluded due to device loss or malfunction, noncompliance, or data errors, and another 49 were excluded for having fewer than three valid days of accelerometer wear time. For participants who did not have 7 days of valid wear, the total minutes of MVPA were adjusted according to the number of valid days they had. Ultimately, we acquired a subset of PA monitoring data comprising 425 participants (197 men, 189 women) who had at least three valid days of accelerometer data from the KNHANES VII-2 2017 dataset, which was accessed for research purposes on February 15th 2023.*

***Fig 1. Flow chart utilized to identify eligible male and female participants for the study.***

*KNHANES: Korea National Health and Nutritional Examination Survey, PA: physical activity*

- Figure 1:

- Lines 381-382: *This study included a moderately sized group of participants, with 425 of the initial 491 invitees providing usable data.*

**14. Lines 310 and 311, please insert something that states "even according to national Guidelines" after the ";" to read "results; XXXXX, subjective PA prevalence ...", so that the reader can grasp that are not just 1 study against another, but rather national guidelines/surveys/whatnot. Are 2010 and 2011 the most recent that indicate for this?;**

**Our Response:**

Thank you for your valuable comment. In accordance with your suggestion, we have added a brief mention of national guidelines in the revised manuscript. We also reviewed more recent guidelines and survey-based studies and noted that, to the best of our knowledge, no substantial updates have been published that focus specifically on the discrepancy between subjective and objective PA measurements in older adults. Moreover, the guidelines in question (issued around 2010/2011) do not explicitly differentiate between varying measurement methods, and as a result, adherence rates can differ markedly depending on whether subjective or objective tools are employed. This discrepancy has been noted in various studies.

In our research context, such discrepancies may appear more pronounced because different studies use varying cut-off values for accelerometer data, which directly influence adherence rates to PA guidelines. This point is further supported by our findings in the domestic older adult population, where the adherence rate changes depending on the cutoff values used.

Because the main goals of our study are (1) to evaluate adherence to PA guidelines among older adults in Korea and (2) to explore factors associated with this adherence, conducting a systematic literature review on discrepancies between measurement methods would extend beyond the scope of our research. Therefore, we plan to conclude our discussion by reinforcing the literature’s findings that such discrepancies have been reported in various studies.

In order to capture the latest research trends, we have incorporated additional recent references that further illustrate this issue. The specific changes made in the manuscript are as follows:

- Lines 384-388: *There have been studies on PA measurement using both subjective and objective methods in older adults with contradictory results; even according to national guidelines, the actual adherence rates can differ significantly depending on the measurement method. For example, subjective PA prevalence was much higher than objective PA in many studies [21,30,45,46], whereas the reverse was observed in others [47,48].*

References:

[21] Shiroma EJ, Cook NR, Manson JE, Buring JE, Rimm EB, Lee I-M. Comparison of Self-Reported and Accelerometer-Assessed Physical Activity in Older Women. PLoS One. 2015;10: e0145950.

**(newly added)** [30] Sallis JF, Saelens BE. Assessment of physical activity by self-report: status, limitations, and future directions. Res Q Exerc Sport. 2000;71: S1-14.

[45] Tucker JM, Welk GJ, Beyler NK. Physical Activity in U.S. Adults: Compliance with the Physical Activity Guidelines for Americans. Am J Prev Med. 2011;40: 454–461.

**(newly added)** [46] Buszkiewicz J, Rose C, Gupta S, Ko LK, Mou J, Moudon AV, et al. A cross-sectional analysis of physical activity and weight misreporting in diverse populations: The Seattle Obesity Study III. Obes Sci Pract. 2020;6: 615–627.

[47] Hurtig-Wennlöf A, Hagströmer M, Olsson LA. The International Physical Activity Questionnaire modified for the elderly: aspects of validity and feasibility. Public Health Nutr. 2010;13: 1847–1854.

**(newly added)** [48] Quinlan C, Rattray B, Pryor D, Northey JM, Anstey KJ, Butterworth P, et al. The accuracy of self-reported physical activity questionnaires varies with sex and body mass index. PLoS One. 2021;16: e0256008.

**15. Why would, from your standpoint as researchers, and on line 321,** **more clinical/biochemical variables better associate with accelerometer (objective) measures? In the same manner, in line 325 to 332, you state findings, but seems to me that some discussion is needed: "These observations indicate that accelerometer-based, objectively measured PA, might reflect a different behavioral landscape than that reflected by selfreported measures". Which landscape? And why? Provide some food for thought!;**

**Our Response:**

Thank you very much for your insightful comment. We agree that a deeper discussion is warranted regarding the phenomenon in which more clinical and biochemical variables tend to show stronger associations with accelerometer-based (objective) measures. We briefly addressed part of this point in our response to Comment #8 above, and we would be pleased to elaborate further on this matter here. From our standpoint as researchers, one of the key reasons for this phenomenon is “subjectivity” in self-report measures. We would like to offer the following interpretation from a statistical signal-to-noise perspective:

1. Influence of Subjectivity in Self-Report Measures

Survey data inevitably contain additional “noise” stemming from respondents’ subjective perceptions. This can be referred to by various names, such as recall bias, social desirability bias, and so on. For instance, some participants tend to overestimate their PA, while others tend to underestimate it, creating additional variations that can mask or weaken true associations with clinical or biochemical outcomes. In contrast, accelerometer data objectively capture movement without this subjective noise, resulting in cleaner signals that more readily reveal significant correlations (i.e., more significant clinical/biochemical variables).

2. Subjectivity as a Potential Confounder

We observed that education emerged as highly significant when using self-reported data in Table 3. This finding suggests that higher levels of education correlate with greater adherence to PA guidelines. However, when accelerometer data were used, this relationship not only lost its statistical significance but also showed a reversed trend, with the OR falling below 1 in most cases (e.g., Model 2 in Table 4 and Models 1–2 in Table 5). This discrepancy could possibly reflect differences in participants’ tendencies to over- or under-report their PA. For example, the results in Table 2 of the study by Buszkiewicz et al. (2020) show that individuals with higher levels of education tend to report higher PA adherence on questionnaires—although they did not use the exact same GPAQ criteria—than accelerometer data indicate [46]. This subjectivity can be further supported by the observation that the activity limitation covariate, which is measured by questionnaire, also emerges as significant in the questionnaire data (Table 3) but not in the accelerometer data (Tables 4–5).

In accelerometer data, such confounding from subjectivity is structurally eliminated, offering a more accurate representation of true PA. In summary, in terms of the “behavioral landscape,” we can suggest that questionnaire data on PA adherence include respondents’ subjective judgments, whereas accelerometer data do not.

3. Implications for Aggregate vs. Correlational Analyses

When estimating overall PA adherence rates across a large population, individual over-reporting and under-reporting can partially cancel each other out, allowing for a reasonably reliable approximation of adherence rate. However, in correlational analyses—where noise in the outcome (i.e., PA adherence measure) directly impacts the significance of covariates—this subjective noise becomes more problematic (here, we use “subjective noise” to mean statistical variation rather than implying that participants’ biases are problematic). It can obscure genuine associations with objective variables, while simultaneously allowing certain covariates (such as education) to appear more significant because they capture the subjective bias itself. Consequently, from a correlational perspective, accelerometer data, which are free from the confounding influence of respondent subjectivity, tend to have an absolute advantage over questionnaire data.

Again, we appreciate your suggestion to provide more context and food for thought, and have accordingly expanded the Discussion section as follows:

- Lines 389-439: *In this study, a noteworthy observation was the discrepancy in adherence rates when measured using self-reported questionnaires versus accelerometer readings. First, the PA adherence rate derived from accelerometer data was directly influenced by the cutoff values. Moreover, analysis of correlates revealed that each measurement method produced different significant covariates. Our analysis suggests that one of the main reasons for this discrepancy is the inherent subjectivity of self-report measures. Survey data, by their nature, introduce additional “noise” stemming from respondents’ subjective perceptions—often referred to as recall bias, social desirability bias, and similar phenomena. More specifically, some participants may overestimate their PA, while others underestimate it, thus creating additional variations that can obscure or weaken the true associations with objective outcomes. Accelerometer data can capture PA objectively, free from subjective noise, resulting in cleaner signals. For instance, in the accelerometer data, not only age and sex—considered significant factors for PA—but also various biochemical and clinical variables such as hypertriglyceridemia, low HDL cholesterol, and diabetes were identified as significant covariates (e.g., Tables 4 and 5). However, with the exception of hypercholesterolemia, these associations did not appear in the questionnaire data (Table 3), indicating that self-reported measures failed to detect such relationships.*

*Furthermore, we observed that education emerged as highly significant when using self-reported data in Table 3. This finding suggests that higher levels of education correlate with greater adherence to PA guidelines. However, when accelerometer data were used, this relationship not only lost its statistical significance but also showed a reversed trend, with the OR falling below 1 in most cases (e.g., Model 2 in Table 4 and Models 1–2 in Table 5). This discrepancy could possibly reflect differences in participants’ tendencies to over- or under-report their PA. For example, the results in Table 2 of the study by Buszkiewicz et al. (2020) show that individuals with higher levels of education tend to report higher PA adherence on questionnaires—although they did not use the exact same GPAQ criteria—than accelerometer data indicate [46]. This subjectivity can be further supported by the observation that the activity limitation covariate, which is measured by questionnaire, also emerges as significant in the questionnaire data (Table 3) but not in the accelerometer data (Tables 4–5).*

*When estimating overall PA adherence rates across a large population, individual over-reporting and under-reporting can partially cancel each other out, allowing for a reasonably reliable approximation of adherence rate. However, in correlational analyses—where noise in the outcome (i.e., PA adherence measure) directly impacts the significance of covariates—this subjective noise becomes more problematic. It can obscure genuine associations with objective variables, while simultaneously allowing certain covariates (such as education) to appear more significant because they capture the subjective bias itself. Consequently, from a correlational perspective, accelerometer data, which are free from the confounding influence of respondent subjectivity, tend to have an absolute advantage over questionnaire data.*

*Building on the perspective described above, we offer the following recommendations, which align with the two main objectives of this study. The first objective is to examine PA adherence among older adults in Korea by employing both self-report questionnaires and accelerometer measurements. For this objective, in cases where accelerometer cutoff values calibrated by a specialized lab are not available, we recommend drawing on both questionnaire-based and accelerometer-based data, rather than prioritizing one over the other. Although questionnaires inherently include subjective noise, the averaging process can mitigate some of its effects, and standardized questionnaire formats are recognized and used worldwide. By contrast, accelerometer data may introduce direct bias in PA adherence rates unless cutoff values appropriate to the population are applied. Therefore, in absence of a laboratory-calibrated cutoff threshold, accelerometer data do not necessarily hold an absolute advantage over questionnaire data. We also do not endorse any specific accelerometer cutoff value simply because it yields, on average, results comparable to those obtained from questionnaire data.*

*On the other hand, for the second objective—investigating factors associated with PA adherence using both measurement tools—we recommend placing greater emphasis on accelerometer data. In our correlates analysis, we found that subjectivity issues directly affect the interpretation of the variables; therefore, accelerometer data offer an absolute advantage in this context.*

Additional references:

[46] Buszkiewicz J, Rose C, Gupta S, Ko LK, Mou J, Moudon AV, et al. A cross-sectional analysis of physical activity and weight misreporting in diverse populations: The Seattle Obesity Study III. Obes Sci Pract. 2020;6: 615–627.

**16. Not really sure what the authors mean by the statement on line 352 "Moreover, the corresponding threshold setting is applicable to the older population in Korea". Applicable how? Is there a particular characteristic that makes it so? Previous data? Literature pointing in that direction? (this does back to my previous comment on why these tresholds).**

**Thank you for allowing me to review your work and looking forward for your reply!**

**Our Response:**

Thank you for the comment regarding the statement on line 352. We agree with the reviewer’s concern regarding the preference and recommendation for a specific accelerometer cutoff value in a situation where no population-specific calibrated cutoff values have been established by a specialized laboratory equipped to assess PA. Although there is another measure of adherence to PA guidelines, namely questionnaires, this method is limited by the subjectivity of the respondents and therefore cannot serve as a standard for accelerometer cutoff values. After careful reconsideration, we decided to remove the statement from the manuscript because it could be misunderstood as implying that the questionnaire data serve as the gold standard.

Following the reviewer’s suggestion, we have chosen to use only thresholds that are documented in previous research and to refrain from making any specific claim about their preference to Korean older adults. Therefore, we now rely solely on thresholds supported in the previous literature (a. 1952 cpm 10-minute bout, b. 1952 cpm 10-minute bout with a 1–2-minute tolerance, c. 1040 cpm 10-minute bout, and d. 1040 cpm 10-minute bout with a 1–2-minute tolerance), without making any specific claim about their applicability to older Koreans. Due to space constraints, the results for a. and d. are presented in the Supporting Information.

We appreciate your feedback, which prompted this revision to maintain methodological clarity and reduce potential misinterpretation. Should you have any further questions or require additional information, we would be glad to elaborate further. The modifications on the revised manuscript are as follows:

- Lines 26-37 (Abstract): ***Results:*** *Self-reported data indicated a 34% adherence rate, whereas accelerometer-based rates ranged from 16% to 62.8%, depending on the cutoff values and tolerance settings. Bland-Altman analyses demonstrated notable differences between subjective and objective measurements. In terms of correlates, the questionnaire data highlighted factors such as education and household income, whereas the accelerometer-based findings emphasized sex, age, hypertriglyceridemia, and low HDL cholesterol.* ***Conclusions:*** *This study revealed substantial discrepancies in both MVPA adherence estimates and significant predictors when comparing self-reported surveys to accelerometer data among older adults. For estimating population-level adherence, in the absence of accelerometer cutoff values established by a specialized lab, both self-reported data and accelerometer measurements offer unique insights. Meanwhile, when analyzing the factors influencing PA adherence, accelerometer data may be preferable, as subjective biases in self-report can affect the observed correlates in statistical results.*
- Lines 82-95: *In this study, we incorporated both the commonly used cutoff level of >1952 cpm for MVPA in middle-aged adults [31–33] and the >1040 cpm threshold derived from a laboratory-based calibration study in an older adult population [27]. In the latter study, the cutoff was established based on oxygen consumption—a representative health indicator of PA—and no significant sex differences were observed in either activity counts or oxygen consumption, thereby eliminating the need for sex-specific criteria. Meanwhile, because the GPAQ generally measures aerobic PA in bouts of at least 10 minutes, PA adherence—defined as achieving ≥150 minutes of MVPA within 7 days—was assessed by summing the total duration of continuous MVPA bouts of 10 minutes or more. In addition, we evaluated an alternative approach that allows a 1–2-minute tolerance when defining 10-minute bouts [34], leading to four scenarios: two cutoffs (1952 cpm and 1040 cpm), each applied with and without the 1–2-minute tolerance.*

*The primary objective of this study is to explore PA adherence among older adults in Korea, using both self-reported questionnaires and accelerometer measurements. The secondary objective is to investigate factors associated with this PA adherence based on both measurement tools.*

- Lines 352-378: *Fig 2 displays Bland-Altman plots for daily MVPA minutes comparing the questionnaire (Q = K-GPAQ) and the accelerometer under four different scenarios: panel A uses a >1952 cpm cutoff (A1952); panel B applies a >1952 cpm cutoff with a 1–2‐minute tolerance (A1952t); panel C uses a >1040 cpm cutoff (A1040); and panel D applies a >1040 cpm cutoff with tolerance (A1040t). The horizontal axis represents the mean of the two measures, as defined in Equation (1), while the vertical axis shows their difference, as specified in Equation (2). In each plot, the dashed central line represents the average difference (bias) across all participants, and the solid lines represent the 95% limits of agreement (± 1.96 SD), calculated using Equation (3). A narrower spread between the solid lines indicates closer agreement, whereas a wider spread suggests greater variability between methods.*

***Fig 2. Bland-Altman plots for daily MVPA minutes comparing the questionnaire data (Q = K-GPAQ) with accelerometer data under the following settings: A. A1952 (>1952cpm). B. A1952t (>1952cpm, tolerated). C. A1040 (>1040cpm). D. A1040t (>1040cpm, tolerated).***

*Panels A and B—which use a higher cutoff (>1952 cpm)—show average differences of 13.81 and 6.72 minutes/day, respectively, with the questionnaire reporting higher MVPA times. In contrast, panels C and D—which use a lower cutoff (>1040 cpm)—exhibit average differences of 2.34 and -15.77 minutes/day, respectively. This implies that applying a higher cutoff criterion for determining 10-minute bouts in the accelerometer data results in a lower recorded MVPA time. Moreover, the mean difference is numerically lower when applying a 1–2 minute tolerance: panel B shows a lower difference than panel A, and panel D a lower difference than panel C. This suggests that even with the same cutoff, incorporating a 1–2 minute tolerance in bout determination tends to increase the recorded MVPA time, as if the cutoff were effectively lowered. Although one combination (panel C, >1040 cpm) exhibited a relatively small overall bias, the triangular boundaries observed in the Bland-Altman plots indicate a pronounced discrepancy between the questionnaire and accelerometer data. These triangular patterns represent cases where one method recorded 0 minutes/day of MVPA: points along the upper boundary correspond to instances where MVPA was reported in the questionnaire but not detected by the accelerometer, whereas points along the lower boundary indicate cases where MVPA was detected by the accelerometer but not reported in the questionnaire.*

- Lines 389-439: *First, the PA adherence rate derived from accelerometer data was directly influenced by the cutoff values. Moreover, analysis of correlates revealed that each measurement method produced different significant covariates. Our analysis suggests that one of the main reasons for this discrepancy is the inherent subjectivity of self-report measures. Survey data, by their nature, introduce additional “noise” stemming from respondents’ subjective perceptions—often referred to as recall bias, social desirability bias, and similar phenomena. More specifically, some participants may overestimate their PA, while others underestimate it, thus creating additional variations that can obscure or weaken the true associations with objective outcomes. Accelerometer data can capture PA objectively, free from subjective noise, resulting in cleaner signals. For instance, in the accelerometer data, not only age and sex—considered significant factors for PA—but also various biochemical and clinical variables such as hypertriglyceridemia, low HDL cholesterol, and diabetes were identified as significant covariates (e.g., Tables 4 and 5). However, with the exception of hypercholesterolemia, these associations did not appear in the questionnaire data (Table 3), indicating that self-reported measures failed to detect such relationships.*

*Furthermore, we observed that education emerged as highly significant when using self-reported data in Table 3. This finding suggests that higher levels of education correlate with greater adherence to PA guidelines. However, when accelerometer data were used, this relationship not only lost its statistical significance but also showed a reversed trend, with the OR falling below 1 in most cases (e.g., Model 2 in Table 4 and Models 1–2 in Table 5). This discrepancy could possibly reflect differences in participants’ tendencies to over- or under-report their PA. For example, the results in Table 2 of the study by Buszkiewicz et al. (2020) show that individuals with higher levels of education tend to report higher PA adherence on questionnaires—although they did not use the exact same GPAQ criteria—than accelerometer data indicate [46]. This subjectivity can be further supported by the observation that the activity limitation covariate, which is measured by questionnaire, also emerges as significant in the questionnaire data (Table 3) but not in the accelerometer data (Tables 4–5).*

*When estimating overall PA adherence rates across a large population, individual over-reporting and under-reporting can partially cancel each other out, allowing for a reasonably reliable approximation of adherence rate. However, in correlational analyses—where noise in the outcome (i.e., PA adherence measure) directly impacts the significance of covariates—this subjective noise becomes more problematic. It can obscure genuine associations with objective variables, while simultaneously allowing certain covariates (such as education) to appear more significant because they capture the subjective bias itself. Consequently, from a correlational perspective, accelerometer data, which are free from the confounding influence of respondent subjectivity, tend to have an absolute advantage over questionnaire data.*

*Building on the perspective described above, we offer the following recommendations, which align with the two main objectives of this study. The first objective is to examine PA adherence among older adults in Korea by employing both self-report questionnaires and accelerometer measurements. For this objective, in cases where accelerometer cutoff values calibrated by a specialized lab are not available, we recommend drawing on both questionnaire-based and accelerometer-based data, rather than prioritizing one over the other. Although questionnaires inherently include subjective noise, the averaging process can mitigate some of its effects, and standardized questionnaire formats are recognized and used worldwide. By contrast, accelerometer data may introduce direct bias in PA adherence rates unless cutoff values appropriate to the population are applied. Therefore, in absence of a laboratory-calibrated cutoff threshold, accelerometer data do not necessarily hold an absolute advantage over questionnaire data. We also do not endorse any specific accelerometer cutoff value simply because it yields, on average, results comparable to those obtained from questionnaire data.*

*On the other hand, for the second objective—investigating factors associated with PA adherence using both measurement tools—we recommend placing greater emphasis on accelerometer data. In our correlates analysis, we found that subjectivity issues directly affect the interpretation of the variables; therefore, accelerometer data offer an absolute advantage in this context.*

- Lines 495-506: *We observed significant demographic variations in PA guideline adherence, including sex, age, hypertriglyceridemia, and low HDL cholesterol levels. However, our study discovered discrepancies between questionnaire data and accelerometer data in analyzing the key factors associated with PA adherence, demonstrating that the potential subjectivity in questionnaire data may introduce issues in interpreting covariates.*

*From the standpoint of policy formulation, it is crucial to rely on objective evidence when assessing PA adherence and identifying its associated covariates. Based on our findings, in evaluating PA adherence rates among the target population (in this case, older adults in Korea), we recommend treating both questionnaire and accelerometer data as equally valid sources, since data reflecting diverse perspectives are essential for developing effective public policies. Meanwhile, we suggest prioritizing accelerometer data when exploring the correlates of PA adherence, given that the inherent subjectivity of questionnaire-based measures more directly affects their interpretation.*

<Reviewer #2>

General Comments

**1. The study brings interesting information about the older Korean adult population, especially regarding PA habits through a study of factors associated with PA engagement.**

**However, some parts of the purpose of this study could be clearer (see details after). The results could be improved by using similar methods along the manuscript. Indeed, in the first part, there are comparisons between questionnaire, accelerometer > 1040 cpm and accelerometer > 1952 cpm whereas in the second part (Bland-Altman plots), the authors compared 1) questionnaire vs accelerometer > 1040 cpm and 2) accelerometer > 1952 cpm with 1 to 2 min tolerance. You could complete by adding information about “accelerometer > 1952 cpm with 1 to 2 min tolerance” in the first part and adding a Bland-Altman plot with “accelerometer > 1952 cpm”.**

**More specifically, the statistical analyses could be refined (see details after).**

**The discussion could be improved. It is not clear what the authors could recommend about the method to use regarding the threshold. In addition, discussing the health factors associated with adherence could add value to the study.**

**Our Response:**

Thank you for your valuable feedback highlighting areas where our study can be strengthened. We will address this in more detail later; for now, we would like to briefly outline the main directions for revising the paper. We address your comments point by point below.

1) Clarification of the Study Purpose

**However, some parts of the purpose of this study could be clearer.**

It is imperative to clarify and articulate the objectives of our research. The primary objective of this study is to explore PA adherence among older adults in Korea using both self-reported questionnaires and accelerometer measurements. The secondary objective is to explore factors associated with PA adherence based on both measurement tools. In the revised manuscript, we have refined our statement of purpose in the Introduction section and updated the title of our paper to “Assessing adherence to physical activity guidelines and correlates among older Korean adults with a focus on 10-minute bout duration using subjective and objective measures,” thereby more accurately reflecting our focus on identifying correlates of adherence.

2) Consistency in Methods and Threshold Comparisons

**The results could be improved by using similar methods along the manuscript. Indeed, in the first part, there are comparisons between questionnaire, accelerometer > 1040 cpm and accelerometer > 1952 cpm whereas in the second part (Bland-Altman plots), the authors compared 1) questionnaire vs accelerometer > 1040 cpm and 2) accelerometer > 1952 cpm with 1 to 2 min tolerance. You could complete by adding information about “accelerometer > 1952 cpm with 1 to 2 min tolerance” in the first part and adding a Bland-Altman plot with “accelerometer > 1952 cpm”.**

Thank you for pointing out the discrepancy in threshold settings of our results. In response, we addressed the following four clear reference-based scenarios in the revised manuscript:

a. 1952 cpm 10-minute bout

b. 1952 cpm 10-minute bout with a 1–2-minute tolerance

c. 1040 cpm 10-minute bout

d. 1040 cpm 10-minute bout with a 1–2-minute tolerance

We have detailed the rationale for using these settings in our responses to Comments #2 and #5. Consequently, to clarify that questionnaire data is not treated as the gold standard, we do not advocate any particular accelerometer cutoff setting as being preferable for older Korean adults. This will help prevent questionnaire data from being misunderstood as a gold standard.

3) Refinement of Statistical Analyses

**More specifically, the statistical analyses could be refined.**

We appreciate your feedback on refining our statistical approach. We have further reinforced the statistical analysis and its interpretation based on your suggestion. Specifically, we introduced “activity limitation” as an additional covariate in the questionnaire items to evaluate potential discrepancies between questionnaire and accelerometer data, and added Model 3 employing LASSO—an advanced statistical method that selects variables while accounting for all interaction terms. (Further details are provided in Comments #10 and #15 below)

4) Discussion of Threshold Recommendations

**The discussion could be improved. It is not clear what the authors could recommend about the method to use regarding the threshold. In addition, discussing the health factors associated with adherence could add value to the study.**

Thank you for the comment. We agree with the reviewer’s concern regarding the Discussion section. Although there is another measure of adherence to PA guidelines, namely questionnaires, this method is limited by the subjectivity of the respondents and therefore cannot serve as a standard for accelerometer cutoff values. After careful reconsideration, we decided not to endorse any single combination (that is, scenarios a. ~ d. above) as preferable for Korean older adults. This decision clarifies that questionnaire data are not being treated as the gold standard. We only address thresholds previously supported in the literature without making any specific claim about their applicability to older Koreans.

We believe that this outline provides a clear overview for interpreting the subsequent responses to the point-by-point comments.

**Specific Comments**

**2. Abstract**

**Adherence to PA guidelines was evaluated using two different thresholds for the accelerometer data.**

**In the results, there are 3 thresholds (1952, 1040 and 560).**

**Our Response:**

Thank you for your comment. The cut points of >1952 cpm [31-33] and >1040 cpm [27] were referenced from studies on accelerometer-based physical activity data in middle-aged adults and older adults, respectively. However, the >560 cpm threshold was arbitrarily set for exploratory purposes. In response to the reviewer’s comment (and considering Comment #14, which implies that this >560 cpm threshold should be removed), we have decided to include only the reference-based cut points documented in the literature and to incorporate a 1–2-minute tolerance (e.g., [34]) when defining a 10-minute bout. As a result, when addressing the accelerometer data, we focus on four clear scenarios:

a) 1952 cpm 10-minute bout

b) 1952 cpm 10-minute bout with a 1–2-minute tolerance

c) 1040 cpm 10-minute bout

d) 1040 cpm 10-minute bout with a 1–2-minute tolerance.

The modifications on the revised manuscript are as follows:

- Lines 24-26 (Abstract): *Adherence to PA guidelines was evaluated using two different thresholds for the accelerometer data, each applied both strictly and with a tolerance of one to two minutes when defining a 10-minute bout.*

References:

[27] Copeland JL, Esliger DW. Accelerometer Assessment of Physical Activity in Active, Healthy Older Adults. J Aging Phys Act. 2009;17: 17–30.

[31] Gorman E, Hanson HM, Yang PH, Khan KM, Liu-Ambrose T, Ashe MC. Accelerometry analysis of physical activity and sedentary behavior in older adults: a systematic review and data analysis. Eur Rev Aging Phys Act. 2014;11: 35–49.

[32] Sun F, Norman IJ, While AE. Physical activity in older people: a systematic review. BMC Public Health. 2013;13: 449.

[33] Jefferis BJ, Sartini C, Lee I-M, Choi M, Amuzu A, Gutierrez C, et al. Adherence to physical activity guidelines in older adults, using objectively measured physical activity in a population-based study. BMC Public Health. 2014;14: 382.

[34] Troiano RP, Berrigan D, Dodd KW, Mâsse LC, Tilert T, Mcdowell M. Physical Activity in the United States Measured by Accelerometer. Med Sci Sports Exercise. 2008;40: 181–188.

**3. Financial disclosure**

**If possible, enter the initials of the authors who received the awards and the URL of the funders’ website. Please, add the statement informing if the funders played any role in the study.**

**Our Response:**

Thank you for your comment. In accordance with the editor’s guidance, we have included the initials of the author who received the funding, the funder’s website (<https://www.nrf.re.kr>), and funder’s role in the coverletter. We also clarified that the funders had no role in the study design, data collection and analysis, decision to publish, or preparation of the manuscript. The modifications on the revised manuscript are as follows:

- Lines 659-662 (Funding): *This work was supported by the National Research Foundation of Korea (NRF) grant funded by the Korea government (MSIT) (No. 2022R1A5A8023404), awarded to C.H.L. The funders’ website is available at* [*https://www.nrf.re.kr*](https://www.nrf.re.kr)*. The funders had no role in the study design, data collection and analysis, decision to publish, or preparation of the manuscript.*

**4. Data availability**

**The URL provided to access the data leads to a blank page (accessed on 22nd January 2025). Please, update the link.**

**Our Response:**

We regret any inconvenience this may cause and appreciate your understanding. We agree that a clearly defined link for data provision is necessary. We apologize that we cannot provide a direct link that takes you to the exact page (<https://knhanes.kdca.go.kr/knhanes/rawDataDwnld/rawDataDwnld.do>), as copying the link from that location does not open the site properly. After contacting the Korea Disease Control and Prevention Agency (KDCA) by phone and requesting a usable link, they provided a rather lengthy, temporary URL but informed us that it would expire after a certain period (<https://knhanes.kdca.go.kr/knhanes/postSendPage.do?url=%2FrawDataDwnld%2FrawDataDwnld.do&postparam=%7B%22menuId%22%3A%20%2210031001%22%7D>). Indeed, it eventually stopped working, and thus we regretfully must include this explanation. Details for the two datasets can be found on the KNHANES website as follows (we additionally ask for your understanding that, due to server instability, even the method we have confirmed to be working may eventually become unavailable):

1) Go to <https://knhanes.kdca.go.kr/knhanes/eng/main.do>
2) Click ‘Survey Data’
3) Click ‘Data Introduction’

The modifications on the revised manuscript are as follows:

- Lines 651-653 (Data availability statement): *The datasets analyzed for this study can be found in the seventh Korea National Health and Nutrition Examination Survey (KNHANES VII-2), 2017, Korea Disease Control and Prevention Agency.* [*https://knhanes.kdca.go.kr/knhanes/eng/main.do*](https://knhanes.kdca.go.kr/knhanes/eng/main.do) *under Survey Data → Data Introduction.*

**5. Introduction**

**Line 88 – 89: I may miss something but I don’t understand why you would like to get a more accurate alignment with questionnaire data as it was a gold-standard fir PA assessment. When reading the paper, it seems you compared accelerometer assessment with different parameters and you found that one is more aligned than the other one. Please rephrase.**

**Our Response:**

Thank you for your insightful comment. We agree with the reviewer’s concern regarding the preference and recommendation for a specific accelerometer cutoff value in a situation where no population-specific calibrated cutoff values have been established by a specialized laboratory equipped to assess PA. Although there is another measure of adherence to PA guidelines, namely questionnaires, this method is limited by the subjectivity of the respondents and therefore cannot serve as a standard for accelerometer cutoff values. After careful reconsideration, we decided to remove the statement from the manuscript because it could be misunderstood as implying that the questionnaire data serve as the gold standard.

Following the reviewer’s suggestion, we have chosen to use only thresholds that are documented in previous research and to refrain from making any specific claim about their preference to Korean older adults. Therefore, we now rely solely on thresholds supported in the previous literature (a. 1952 cpm 10-minute bout, b. 1952 cpm 10-minute bout with a 1–2-minute tolerance, c. 1040 cpm 10-minute bout, and d. 1040 cpm 10-minute bout with a 1–2-minute tolerance), without making any specific claim about their applicability to older Koreans. Due to space constraints, the results for a. and d. are presented in the Supporting Information.

We appreciate your feedback, which prompted this revision to maintain methodological clarity and reduce potential misinterpretation. Should you have any further questions or require additional information, we would be glad to elaborate further. The modifications on the revised manuscript are as follows:

- Lines 82-95: *In this study, we incorporated both the commonly used cutoff level of >1952 cpm for MVPA in middle-aged adults [31–33] and the >1040 cpm threshold derived from a laboratory-based calibration study in an older adult population [27]. In the latter study, the cutoff was established based on oxygen consumption—a representative health indicator of PA—and no significant sex differences were observed in either activity counts or oxygen consumption, thereby eliminating the need for sex-specific criteria. Meanwhile, because the GPAQ generally measures aerobic PA in bouts of at least 10 minutes, PA adherence—defined as achieving ≥150 minutes of MVPA within 7 days—was assessed by summing the total duration of continuous MVPA bouts of 10 minutes or more. In addition, we evaluated an alternative approach that allows a 1–2-minute tolerance when defining 10-minute bouts [34], leading to four scenarios: two cutoffs (1952 cpm and 1040 cpm), each applied with and without the 1–2-minute tolerance.*

*The primary objective of this study is to explore PA adherence among older adults in Korea, using both self-reported questionnaires and accelerometer measurements. The secondary objective is to investigate factors associated with this PA adherence based on both measurement tools.*

**6. Statistical analysis**

**Line 113: results related to 560 are not showed in the document.**

**Our Response:**

Thank you for your comment. As mentioned in Comments #2 and #5 above, the 560 cpm has been removed from the revised manuscript, and consequently, no results for 560 cpm are presented.

**7. Line 117 – 121: Why didn’t you consider to use the “less stringent criterion” in the descriptive results and in the logistic models?**

**Our Response:**

Thank you for your comment. As detailed in Comments #2 and #5 above, all possible cases referenced in the literature (that is, a ~ d scenarios) have been taken into account in both the descriptive results and the logistic models. Due to space limitations, only the logistic regression results for the >1952 cpm cutoff (no tolerance) and for the >1040 cpm cutoff with tolerance are presented in Tables S1 and S2 in the Supporting Information. The specific revisions made to the manuscript are as follows:

- Lines 154-174: *One such proposed definition involves a 10-minute bout with an allowance for interruptions of up to 1–2 minutes [34]. This less stringent criterion recognizes sporadic disruptions that often occur in everyday life, and enables a more realistic and comprehensive understanding of an individual's engagement in MVPA. Consequently, four scenarios were considered using two cutoffs (1952 cpm and 1040 cpm), each assessed with and without the 1–2-minute tolerance.*

***Physical activity adherence***

*PA adherence was defined as engaging in 150 to 300 minutes of moderate-intensity aerobic PA or at least 75 to 150 minutes of vigorous-intensity aerobic PA, or an equivalent combination of moderate- and vigorous-intensity activity throughout the week, as measured using K-GPAQ. For the accelerometer-based assessment, we analyzed four distinct scenarios to capture MVPA bouts of at least 10 consecutive minutes, differentiated by cutoff values (1952 cpm or 1040 cpm) and the allowance of a brief tolerance period (1–2 minutes) within each bout. Specifically, the four scenarios are:*

*a) 1952 cpm 10-minute bout: Participants must maintain ≥1952 counts per minute for the full 10-minute period.*

*b) 1952 cpm 10-minute bout with a 1–2-minute tolerance: Participants must maintain ≥1952 cpm for at least 8 out of 10 minutes, permitting up to 2 minutes below the threshold.*

*c) 1040 cpm 10-minute bout: Participants must maintain ≥1040 cpm for the entire 10-minute interval.*

*d) 1040 cpm 10-minute bout with a 1–2-minute tolerance: Participants must maintain ≥1040 cpm for at least 8 out of 10 minutes, allowing up to 2 minutes below the threshold.*

*If the total MVPA minutes computed from the accelerometer reached or exceeded 150 minutes per week under any scenario, the participant was considered to have adhered to the PA guidelines.*

- Line 245 (Table 1): We found that the lower section of Table 1 (PA adherence) in the original manuscript and the upper section of Table 2 contained identical content and test results. Therefore, to avoid redundancy, we removed the lower section of Table 1.
- Lines 260-261 (Table 2): We have added the less stringent criterion (tolerance) in the descriptive results.
- Lines 275-279: *In this section, we address the correlates of PA guidelines adherence among older adults using three measurement approaches: (1) self‐reported data from the K‐GPAQ, (2) accelerometer data with a >1952 cpm cutoff and a 1–2‐minute tolerance, and (3) accelerometer data with a >1040 cpm cutoff.* *Due to space limitations, results for the >1952 cpm cutoff (no tolerance) and for the >1040 cpm cutoff with tolerance are presented in Tables S1 and S2 in the Supporting Information.*

**8. Line 162, 163: Please add a R citation in the text as recommended by the software providers.**

**Our Response:**

Thank you for your valuable comment regarding the citation for the software providers. We recognize the importance of properly acknowledging the software used in our research. In response to the reviwer’s suggestion, we have revised our manuscript to include appropriate citations in the text and updated the references section accordingly. We appreciate your guidance on this matter and believe that these changes will help improve the clarity and integrity of our work. The modifications on the revised manuscript are as follows:

- Lines 204-208: *Statistical analysis was performed using SAS version 9.4 [37], R version 4.4.2 [38], and additional R packages. Specifically, the dplyr package was utilized for data preprocessing [39], the car package for assessing multicollinearity [40], the mice package for applying multiple imputation [41], the glmnet package for LASSO (least absolute shrinkage and selection operator) logistic regression analysis [42,43], and the ggplot2 package for generating Bland-Altman plots [44].*

Additional references:

[37] SAS Institute. Base SAS 9.4 procedures guide: Statistical procedures. 2017.

[38] R Core Team, R, Others. R: A language and environment for statistical computing. 2013. Available: https://www.R-project.org/

[39] Wickham H, François R, Henry L, Müller K, Vaughan D. dplyr: A Grammar of Data Manipulation. 2023. Available: https://dplyr.tidyverse.org

[40] Fox J, Weisberg S. An R companion to applied regression. 2018.

[41] van Buuren S, Groothuis-Oudshoorn K. mice: Multivariate Imputation by Chained Equations in R. Journal of Statistical Software. 2011;45: 1–67.

[42] Friedman J, Tibshirani R, Hastie T. Regularization Paths for Generalized Linear Models via Coordinate Descent. Journal of Statistical Software. 2010;33: 1–22.

[43] Kenneth Tay J, Narasimhan B, Hastie T. Elastic Net Regularization Paths for All Generalized Linear Models. Journal of Statistical Software. 2023;106: 1–31.

[44] Wickham H. ggplot2: Elegant Graphics for Data Analysis. Springer-Verlag New York; 2016.

**9. Line 165: Make sure / check the use of t-tests is valid in each case. It may have been done since you used Welch t-tests in Table 2 (line 220).**

**Our Response:**

We sincerely apologize for any confusion caused by this oversight and appreciate your helpful feedback. We have carefully re-checked our analyses and confirmed that we used the Welch t-test (which is the default method in R) throughout. The mention of a t-test in Table 2 was a mistake, and we have now corrected it to clarify that Welch t-tests were consistently applied.

As you noted, the Welch t-test does not assume equal variances and is therefore more robust across a wider range of conditions. When variances are equal, Welch t-tests produce similar results to the classic t-test; however, if variances differ, the classic t-test can be problematic whereas the Welch t-test remains valid. We appreciate your careful reading and have updated our manuscript as follows:

- Line 247 (Table 1): *^a^Welch's t-tests and chi-square tests were applied to the analysis of continuous and categorical variables, respectively.*
- Line 265 (Table 2): *^c^Welch's t-tests were used to compare continuous variables.*

**10. Line 170: It seems you didn’t consider the interaction factors in Model 2, especially age vs other factors, sex vs other factors, smoking vs hypertension, etc. You should add the significant ones in Models 2 and 3 (see a note hereafter) but the number of factors will increase (cf note below).**

**Our Response:**

Thank you very much for your insightful comments regarding the potential interactions in our analysis. We fully agree that interactions should be examined, as they arise when considering the joint effects of two or more factors and indicate patterns that deviate from simple additivity. Examples include synergy (where the combined effect exceeds the sum of individual factors) and negative synergy (where the combined effect is smaller than expected). Accounting for these interaction terms can lead to a more refined model and richer statistical interpretations.

As you noted in Comment #15, we do have a large number of variables, and may consider removing variables that are non-significant. To that end, we applied LASSO (least absolute shrinkage and selection operator) for variable selection. LASSO incorporates an L1 penalty on regression coefficients, effectively shrinking some coefficients to zero while simultaneously addressing both variable selection and model regularization. This stable and advanced method helps reduce overfitting and improves generalizability.

Following the reviewer’s suggestion, we introduced “Model 3,” which considered all 17 chosen covariates plus their interactions, and then applied LASSO to select only the statistically significant predictors. The model was constructed by first performing a LASSO logistic regression with only first-order terms, selecting 3–5 variables based on the LASSO path. Then, all variables and their their interaction terms were entered into another LASSO analysis, allowing for the inclusion of one possible additional variable. The final model comprises the initially selected variables plus any significant interaction terms (and their main effects if not already included). This procedure not only helps identify significant variables and detect potential interactions, but also provides results that are relatively stable and generalizable.

Although LASSO can be applied in high-dimensional settings directly (that is, entering all variables and their interaction terms into the LASSO analysis from the beginning), in our research context, challenges may arise as sensitivity to such settings can lead to unstable results. Specifically, small changes in the optimization settings can result in drastic variations in the combination of selected variables, making it difficult to generalize. Thus, we used the two-step method mentioned above to detect interactions while producing relatively stable results.

We performed a LASSO path analysis (Model 3) to identify significant variables, and as expected, the results of Model 3 were not much different from those of Models 1 and 2. Also, we found that most interaction terms were not significant, with only one exception. Specifically, in the case of “>1040 cpm, ≥10 minute bouts with 1–2 minute tolerance” (Table S2), the interaction between “unmet medical needs” and “activity limitation” reached statistical significance. When these two variables co-occurred, the adherence to PA guideline was substantially lower (odds ratio of 0.07). For your reference, we have provided the following Model 3 subset from Table S2:

| **Model 3 from Table S2** | | |
| --- | --- | --- |
|  | **OR [95% CI]** | **p-value** |
| **Sex, male** | 1.45 [0.90, 2.34] | 0.121 |
| **Marital status** | 1.89 [1.15, 3.12] | **0.013** |
| **Diabetes** | 0.49 [0.31, 0.78] | **0.003** |
| **Low HDL cholesterol** | 0.59 [0.38, 0.92] | **0.018** |
| **Unmet medical needs** | 0.66 [0.29, 1.51] | 0.315 |
| **Activity limitations** | 1.28 [0.66, 2.57] | 0.468 |
| **Unmet * Activity** | 0.07 [0.00, 0.54] | **0.026** |

When the interaction term is not included in the model, the results for the remaining variables remain largely unchanged, although “unmet medical needs” becomes significant while “activity limitation” does not:

| **The case where the interaction is removed** | | |
| --- | --- | --- |
|  | **OR [95% CI]** | **p-value** |
| **Sex, male** | 1.50 [0.93, 2.41] | 0.092 |
| **Marital status** | 1.90 [1.16, 3.13] | **0.011** |
| **Diabetes** | 0.50 [0.31, 0.79] | **0.003** |
| **Low HDL cholesterol** | 0.60 [0.39, 0.93] | **0.021** |
| **Unmet medical needs** | 0.38 [0.19, 0.77] | **0.008** |
| **Activity limitations** | 0.91 [0.50, 1.66] | 0.754 |

Our interpretation is as follows: generally, individuals reporting “activity limitation” do not exhibit a distinct pattern in their adherence to “>1040 cpm, ≥10 minute bouts with 1–2 minute tolerance.” However, those who have both “unmet medical needs” and “activity limitation” show a pronounced decrease in adherence, indicating that these two variables act synergistically to reduce PA adherence.

We would like to emphasize that caution is warranted in interpreting interactions. While some studies may consistently report specific interactions (e.g., APOE4 allele by sex), in general, interactions are relatively rare. Also, exploring two-way interactions among 17 variables generates _17_C_2_=136 possible combinations, increasing the likelihood of spurious findings by chance. Since identifying covariates is an exploratory analysis, we did not perform adjustments for multiple testing. Therefore, rather than drawing premature conclusions, we believe it is prudent to await future studies to confirm or refute our observation. Because of space constraints and concern that overemphasizing a potentially chance finding might confuse readers, we have chosen to include this interaction result in the Supporting Information only. If you feel that a more prominent discussion of these findings is warranted in the main text, we would be happy to incorporate it.

In summary, we have kept Models 1 and 2 as they are and added Model 3 using the LASSO variable selection. We found no significant interactions in most cases, and we placed the single significant interaction (between “unmet medical needs” and “activity limitation” under the specified conditions) in Table S2 of the Supporting Information.

Thank you again for your valuable suggestions, and please let us know if you have any further concerns or comments. We are grateful for the opportunity to refine our manuscript in light of your feedback. The modifications on the revised manuscript are as follows:

- Lines 217-223: *Model 3 employed a two-step LASSO logistic regression approach: first, only first-order terms were considered, selecting three to five variables based on the LASSO path; second, all variables along with their interaction terms were entered into another LASSO analysis, allowing for the inclusion of one possible additional variable. The final model thus comprised those initially selected variables plus any significant interaction terms (and their main effects if not already included). This multi-model strategy enabled us to explore both minimal and comprehensive covariate adjustments, as well as to systematically identify potentially important interactions in a data-driven manner.*
- Lines 300-304: *Finally, Model 3, which was derived using a two‐stage LASSO selection procedure, highlighted the following set of variables—education (OR=1.32 [1.08, 1.60]), standardized household income (OR=1.26 [1.02, 1.57]), and hypercholesterolemia (OR=1.73 [1.10, 2.71]) as positive correlates; hypertension (OR=0.60 [0.39, 0.93]) and activity limitations (OR=0.45 [0.21, 0.89]) as negative correlates.*
- Lines 313-319: *Finally, Model 3 yielded a more parsimonious selection of significant predictors, retaining sex (OR=2.23 [1.40, 3.57]) while identifying two lipid‐related factors—hypertriglyceridemia (OR=0.30 [0.10, 0.74]) and low HDL (OR=0.39 [0.09, 0.56])—as significant negative correlates. Although unmet medical needs and economic activity were selected during the LASSO variable selection process, they did not reach statistical significance in the final model constructed with the chosen variables. It is worthwhile noting that a non-significant result does not imply the absence of an effect; the corresponding OR values can remain meaningful.*
- Lines 335-336: *Finally, in Model 3, male (OR=2.19 [1.44, 3.34]), diabetes (OR=0.60, [0.36, 0.97]), and low HDL (OR=0.41 [0.26, 0.64]) emerged as significant predictors.*
- Lines 454-456: *In the logistic regression analysis, we explored a potential interaction using LASSO in Model 3. For the most part, the main findings did not reveal any significant interactions (Tables 3–5). However, when applying accelerometer data with a >1040 cpm cutoff, ≥10-minute bouts, and a 1–2-minute tolerance (Table S2), we detected significant interactions involving unmet medical needs and activity limitation. Specifically, although activity limitation on its own was not significant, it exerted a synergistic effect when combined with unmet medical needs, resulting in a substantial decrease in PA adherence. In general, it is relatively uncommon to observe an interaction effect. Moreover, given the large number of potential interaction terms tested, this finding may have arisen by chance (i.e., multiple testing). Hence, it should be interpreted with caution, and further research is required to confirm it.*
- Tables 3-5: Model 3 has been added to each table.

**11. Line 172: “the primary analyses were replicated using different thresholds”. Only one threshold is written. Please specify the thresholds you used.**

**Our Response:**

Thank you for the comment. Following the reviewer’s suggestion, we specified all of the thresholds used in the analysis as follows:

- Lines 224-225: *Sensitivity analyses were conducted, where the primary analyses were replicated using different criteria (i.e., scenarios a to d in the Physical activity adherence subsection) for defining MVPA.*

**12. Line 179: “accelrometer” -> “accelerometer” (typo).**

**Our Response:**

Thank you for the comment. Following the reviewer’s suggestion, we have corrected the typo as follows:

- Line 232:

$$Difference=K-GPAQ minutes of MVPA per day - accelerometer minutes of MVPA per day.$$

**13. Results**

**Line 186: “the study’s participation process” (to be rephrased).**

**Our Response:**

Thank you for the comment. We would like to clarify that we underwent extensive revisions during the review process, and as a result, Figure 1 and its descriptions were moved to the Sample subsection of the Materials and Methods section. Also, the corresponding sentence has been rephrased as follows:

- Lines 114-116: *Fig 1 presents the selection process used to derive our analytic sample from the whole dataset, illustrating how older adults (≥65 years) meeting the study’s inclusion criteria were identified and further filtered based on accelerometer-based PA data availability.*

**14. Line 208 and Table 2: Why didn’t you consider the 560 cpm threshold? This threshold should probably be removed from the abstract and the methods.**

**Our Response:**

Thank you for your comment. As mentioned in Comments #1 and #5, we have decided to remove the 560 cpm threshold throughout this paper. Hence, the 560 cpm threshold has been removed from the abstract, methods, and all related analyses, ensuring consistency across the manuscript.

**15. Line 229 (Table 3), Line 257 (Table 4), Line 272 (Table 5): Model 2 involves many factors. The conditions for applying logistic regression may not be met since there only 133 participants with adherence (for Table 3). Suggestion: You might consider a Model 3 involving only factors with a p value less than a threshold (p < 0.2 for instance), such as “Drinking alcohol” (in Table 3). However, I don’t think your conclusions will be changed.**

**Our Response:**

Thank you for your insightful comment. We acknowledge that Model 2 includes a considerable number of variables, which may result in a slight deviation from the generally recommended events-per-variable (EPV) guideline of approximately 10 events per predictor. In particular, given there are only 133 participants classified as adherent in this study, the EPV may be somewhat lower than the ideal level. This provides motivation for the selection of variables for analysis.

A threshold-based variable selection approach (e.g., p < 0.2) is indeed a valid method, and we recognize its practical merits. At the same time, changing the order in which variables are evaluated can lead to different final outcomes—even with the same dataset—raising questions about reproducibility and transparency. By contrast, LASSO tends to be more stable, and the LASSO path allows us to clearly disclose any potential changes that occur during the variable selection, thereby enhancing clarity. In the R code we are making public, the variable selection process and the function for drawing the LASSO path are both fully incorporated. For these reasons, we have chosen to employ LASSO as our variable selection method.

With respect to the point raised by the reviewer, we introduced a Model 3 using LASSO logistic regression to address both variable selection and model regularization. In implementing Model 3, we first performed LASSO logistic regression using only first-order terms, selecting a set of key predictors from the full list. Next, we included all variables and their interaction terms in a second LASSO analysis to capture any additional important factors. Ultimately, this process yielded a refined model containing the most significant variables (and the relevant interaction term mentioned in Comment #10).

For comparison purposes, we also retained Model 2, as including all candidate variables in a single model allows us to observe how each variable influences adherence when all other factors are taken into account. Examining both models confirms that our main conclusions remain consistent, even though Model 3 is more parsimonious.

We appreciate your suggestion and believe that this approach both addresses your concerns and preserves the primary conclusions of our study. We hope the additional model will provide greater clarity and methodological rigor, and we are happy to provide additional details or analyses if needed. The modifications on the revised manuscript are as follows:

- Lines 217-223: *Model 3 employed a two-step LASSO logistic regression approach: first, only first-order terms were considered, selecting three to five variables based on the LASSO path; second, all variables along with their interaction terms were entered into another LASSO analysis, allowing for the inclusion of one possible additional variable. The final model thus comprised those initially selected variables plus any significant interaction terms (and their main effects if not already included). This multi-model strategy enabled us to explore both minimal and comprehensive covariate adjustments, as well as to systematically identify potentially important interactions in a data-driven manner.*
- Lines 300-304: *Finally, Model 3, which was derived using a two‐stage LASSO selection procedure, highlighted the following set of variables—education (OR=1.32 [1.08, 1.60]), standardized household income (OR=1.26 [1.02, 1.57]), and hypercholesterolemia (OR=1.73 [1.10, 2.71]) as positive correlates; hypertension (OR=0.60 [0.39, 0.93]) and activity limitations (OR=0.45 [0.21, 0.89]) as negative correlates.*
- Lines 313-319: *Finally, Model 3 yielded a more parsimonious selection of significant predictors, retaining sex (OR=2.23 [1.40, 3.57]) while identifying two lipid‐related factors—hypertriglyceridemia (OR=0.30 [0.10, 0.74]) and low HDL (OR=0.39 [0.09, 0.56])—as significant negative correlates. Although unmet medical needs and economic activity were selected during the LASSO variable selection process, they did not reach statistical significance in the final model constructed with the chosen variables. It is worthwhile noting that a non-significant result does not imply the absence of an effect; the corresponding OR values can remain meaningful.*
- Lines 335-336: *Finally, in Model 3, male (OR=2.19 [1.44, 3.34]), diabetes (OR=0.60, [0.36, 0.97]), and low HDL (OR=0.41 [0.26, 0.64]) emerged as significant predictors.*
- Lines 454-462: *In the logistic regression analysis, we explored a potential interaction using LASSO in Model 3. For the most part, the main findings did not reveal any significant interactions (Tables 3–5). However,* *when applying accelerometer data with a >1040 cpm cutoff, ≥10-minute bouts, and a 1–2-minute tolerance (Table S2), we detected significant interactions involving unmet medical needs and activity limitation. Specifically, although activity limitation on its own was not significant, it exerted a synergistic effect when combined with unmet medical needs, resulting in a substantial decrease in PA adherence. In general, it is relatively uncommon to observe an interaction effect. Moreover, given the large number of potential interaction terms tested, this finding may have arisen by chance (i.e., multiple testing). Hence, it should be interpreted with caution, and further research is required to confirm it.*
- Tables 3-5: Model 3 has been added to each table.

**16. Table 5 vs Fig. 2: The result message may be clearer if the criteria are similar, e.g. “1952 cpm, bouts lasting ≥ 10 min” or “1952 cpm, bouts lasting ≥ 10 min with 1 to 2 min tolerance”.**

**Our Response:**

Thank you for the comment. Following the reviewer’s suggestion, we have chosen to use similar criteria, resulting in four clear reference-based scenarios as follows:

a) 1952 cpm 10-minute bout

b) 1952 cpm 10-minute bout with a 1–2-minute tolerance

c) 1040 cpm 10-minute bout

d) 1040 cpm 10-minute bout with a 1–2-minute tolerance.

We appreciate your suggestion, as this revision will provide greater clarity. The modifications in the revised manuscript are as follows:

- Table 2: We have revised Table 2 to address all scenarios from a to d.
- Tables S1 and S2: We have added the analysis results in S1 Table for scenario a and in S2 Table for scenario d, thereby enabling us to cover all scenarios from a to d.
- Fig 2: We have revised Figure 2 to address all scenarios from a to d.

**17. Lines 285 – 287: You could help the reader to detect what brings the graph in your study.**

**Moreover, your manuscript includes only two graphs. What is the evidence showing that this threshold is optimal for minimizing the bias?**

**Our Response:**

Thank you for your valuable comment. We agree that addressing all four scenarios (mentioned in Comment #16) in the context of the Bland-Altman plots will help clarify the additional insights these scenarios bring to our study.

We have also expanded the description of our Bland-Altman plots, highlighting how readers can interpret the average difference (i.e., bias) between the two measurement methods and the 95% limits of agreement. By examining the distribution of data points around the mean difference line, readers can visually assess where systematic bias might occur and how large the variability is across the range of measurements. We have updated the figure captions and the corresponding Results section to guide readers more clearly through this interpretation.

Our results indicated that certain thresholds—that is, scenarios in b and c Comment #16—tended to show lower bias compared to others. However, as mentioned in Comment #5, we do not endorse any single criterion as being more favorable for older Korean adults. This should clarify that questionnaire data are not treated as the gold standard. Due to space constraints, the model results for scenarios a and d have been included in the Supporting Information. We have revised the manuscript to ensure readers know exactly where to find this information.

We appreciate your suggestion to more clearly demonstrate the interpretation of the Bland-Altman plots and to discuss threshold optimality. The modifications in the revised manuscript are as follows:

- Fig 2: We have revised Figure 2 to address all scenarios from a to d.

- Lines 352-378: *Fig 2 displays Bland-Altman plots for daily MVPA minutes comparing the questionnaire (Q = K-GPAQ) and the accelerometer under four different scenarios: panel A uses a >1952 cpm cutoff (A1952); panel B applies a >1952 cpm cutoff with a 1–2‐minute tolerance (A1952t); panel C uses a >1040 cpm cutoff (A1040); and panel D applies a >1040 cpm cutoff with tolerance (A1040t). The horizontal axis represents the mean of the two measures, as defined in Equation (1), while the vertical axis shows their difference, as specified in Equation (2). In each plot, the dashed central line represents the average difference (bias) across all participants, and the solid lines represent the 95% limits of agreement (± 1.96 SD), calculated using Equation (3). A narrower spread between the solid lines indicates closer agreement, whereas a wider spread suggests greater variability between methods.*

***Fig 2. Bland-Altman plots for daily MVPA minutes comparing the questionnaire data (Q = K-GPAQ) with accelerometer data under the following settings: A. A1952 (>1952cpm). B. A1952t (>1952cpm, tolerated). C. A1040 (>1040cpm). D. A1040t (>1040cpm, tolerated).***

*Panels A and B—which use a higher cutoff (>1952 cpm)—show average differences of 13.81 and 6.72 minutes/day, respectively, with the questionnaire reporting higher MVPA times. In contrast, panels C and D—which use a lower cutoff (>1040 cpm)—exhibit average differences of 2.34 and -15.77 minutes/day, respectively. This implies that applying a higher cutoff criterion for determining 10-minute bouts in the accelerometer data results in a lower recorded MVPA time. Moreover, the mean difference is numerically lower when applying a 1–2 minute tolerance: panel B shows a lower difference than panel A, and panel D a lower difference than panel C. This suggests that even with the same cutoff, incorporating a 1–2 minute tolerance in bout determination tends to increase the recorded MVPA time, as if the cutoff were effectively lowered. Although one combination (panel C, >1040 cpm) exhibited a relatively small overall bias, the triangular boundaries observed in the Bland-Altman plots indicate a pronounced discrepancy between the questionnaire and accelerometer data. These triangular patterns represent cases where one method recorded 0 minutes/day of MVPA: points along the upper boundary correspond to instances where MVPA was reported in the questionnaire but not detected by the accelerometer, whereas points along the lower boundary indicate cases where MVPA was detected by the accelerometer but not reported in the questionnaire.*

**18. Discussion**

**Lines 325 – 332: What are your recommendations to readers? Should we consider that the associated factors are those highlighted with accelerometers, those highlighted with questionnaires, both? This is important to identify the factors on which public policy can focus on to address the lack of PA (cf Line 95).**

**Our Response:**

Thank you for your thoughtful comment. We appreciate the opportunity to clarify our recommendations for readers regarding which factors might be most relevant for public policy interventions addressing PA. Recognizing the importance of grounding public policy in objective evidence, we offer the following considerations from our perspective as researchers.

One of the most significant limitations of self-report measures is their “subjectivity.” We would like to offer the following interpretation from a statistical signal-to-noise perspective regarding this:

1. Influence of Subjectivity in Self-Report Measures

Survey data inevitably contain additional “noise” stemming from respondents’ subjective perceptions. This can be referred to by various names, such as recall bias, social desirability bias, and so on. Specifically, some participants may overestimate their PA, while others underestimate it, creating additional variations that can mask or weaken true associations with objective outcomes such as clinical or biochemical outcomes. In contrast, accelerometer data objectively capture movement without this subjective noise, resulting in cleaner signals that more readily reveal significant correlations (i.e., more significant clinical/biochemical variables).

2. Subjectivity as a Potential Confounder

We observed that education emerged as highly significant when using self-reported data. This finding suggests that higher levels of education correlate with greater adherence to PA guidelines. However, when using accelerometer-based data, this statistical significance was not observed. In most cases (i.e., Model 2 in Table 4 and Models 1-2 in Table 5 of the revised manuscript), the odds ratio actually fell below 1, suggesting an opposite direction. This discrepancy could possibly reflect differences in participants’ tendencies to over- or under-report their PA. For example, the results in Table 2 of the study by Buszkiewicz et al. (2020) show that individuals with higher levels of education tend to report higher PA adherence on questionnaires—although they did not use the exact same GPAQ criteria—than accelerometer data indicate. This subjectivity can be further supported by the observation that the activity limitation covariate, which is measured by questionnaire, also emerges as significant in the questionnaire data (Table 3) but not in the accelerometer data (Tables 4–5).

In accelerometer data, such confounding from subjectivity is structurally eliminated, offering a more accurate representation of true PA. In other words, our findings suggest that questionnaire data on PA adherence include respondents’ subjective judgments, whereas accelerometer data do not.

3. Implications for Aggregate vs. Correlational Analyses

When estimating overall PA adherence rates across a large population, individual over-reporting and under-reporting can partially cancel each other out, allowing for a reasonably reliable approximation of adherence. However, in correlational analyses—where noise in the outcome (i.e., PA adherence measure) directly impacts the significance of covariates—this subjective noise becomes more problematic (here, we use “subjective noise” to mean statistical variation rather than implying that participants’ biases are problematic). It can obscure genuine associations with objective variables, while simultaneously allowing certain covariates (such as education) to appear more significant because they capture the subjective bias itself. Consequently, from a correlational perspective, accelerometer data, which are free from the confounding influence of respondent subjectivity, tend to have an absolute advantage over questionnaire data.

Building on the perspective described above, we offer the following recommendations, which align with the two main objectives of this study. The first objective is to examine PA adherence among older adults in Korea by employing both self-report questionnaires and accelerometer measurements. For this objective, in cases where accelerometer cutoff values calibrated by a specialized lab are not available, we recommend drawing on both questionnaire-based and accelerometer-based data, rather than prioritizing one over the other. Although questionnaires inherently include subjective noise, the averaging process can mitigate some of its effects, and standardized questionnaire formats are recognized and used worldwide. By contrast, accelerometer data may introduce direct bias in PA adherence rates unless cutoff values appropriate to the population are applied. Therefore, in absence of a laboratory-calibrated cutoff threshold, accelerometer data do not necessarily hold an absolute advantage over questionnaire data. We also do not endorse any specific accelerometer cutoff value simply because it yields, on average, results comparable to those obtained from questionnaire data.

On the other hand, for the second objective—investigating factors associated with PA adherence using both measurement tools—we recommend placing greater emphasis on accelerometer data. In our correlates analysis, we found that subjectivity issues directly affect the interpretation of the variables; therefore, accelerometer data offer an absolute advantage in this context.

To summarize, in evaluating PA adherence rates among the target population (in this case, older adults in Korea), we recommend treating both questionnaire and accelerometer data as equally valid sources. This is because data reflecting diverse perspectives are essential for developing effective public policies. Meanwhile, we recommend prioritizing accelerometer data for exploring correlates of PA adherence, given that subjective noise more directly affects the interpretation of questionnaire-based measures. We would like to express our sincere gratitude for your invaluable comments and suggestions, which have greatly contributed to improving the overall quality of our manuscript. The modifications in the revised manuscript are as follows:

- Lines 389-439: *In this study, a noteworthy observation was the discrepancy in adherence rates when measured using self-reported questionnaires versus accelerometer readings. First, the PA adherence rate derived from accelerometer data was directly influenced by the cutoff values. Moreover, analysis of correlates revealed that each measurement method produced different significant covariates. Our analysis suggests that one of the main reasons for this discrepancy is the inherent subjectivity of self-report measures. Survey data, by their nature, introduce additional “noise” stemming from respondents’ subjective perceptions—often referred to as recall bias, social desirability bias, and similar phenomena. More specifically, some participants may overestimate their PA, while others underestimate it, thus creating additional variations that can obscure or weaken the true associations with objective outcomes. Accelerometer data can capture PA objectively, free from subjective noise, resulting in cleaner signals. For instance, in the accelerometer data, not only age and sex—considered significant factors for PA—but also various biochemical and clinical variables such as hypertriglyceridemia, low HDL cholesterol, and diabetes were identified as significant covariates (e.g., Tables 4 and 5). However, with the exception of hypercholesterolemia, these associations did not appear in the questionnaire data (Table 3), indicating that self-reported measures failed to detect such relationships.*

*Furthermore, we observed that education emerged as highly significant when using self-reported data in Table 3. This finding suggests that higher levels of education correlate with greater adherence to PA guidelines. However, when accelerometer data were used, this relationship not only lost its statistical significance but also showed a reversed trend, with the OR falling below 1 in most cases (e.g., Model 2 in Table 4 and Models 1–2 in Table 5). This discrepancy could possibly reflect differences in participants’ tendencies to over- or under-report their PA. For example, the results in Table 2 of the study by Buszkiewicz et al. (2020) show that individuals with higher levels of education tend to report higher PA adherence on questionnaires—although they did not use the exact same GPAQ criteria—than accelerometer data indicate [46]. This subjectivity can be further supported by the observation that the activity limitation covariate, which is measured by questionnaire, also emerges as significant in the questionnaire data (Table 3) but not in the accelerometer data (Tables 4–5).*

*When estimating overall PA adherence rates across a large population, individual over-reporting and under-reporting can partially cancel each other out, allowing for a reasonably reliable approximation of adherence rate. However, in correlational analyses—where noise in the outcome (i.e., PA adherence measure) directly impacts the significance of covariates—this subjective noise becomes more problematic. It can obscure genuine associations with objective variables, while simultaneously allowing certain covariates (such as education) to appear more significant because they capture the subjective bias itself. Consequently, from a correlational perspective, accelerometer data, which are free from the confounding influence of respondent subjectivity, tend to have an absolute advantage over questionnaire data.*

*Building on the perspective described above, we offer the following recommendations, which align with the two main objectives of this study. The first objective is to examine PA adherence among older adults in Korea by employing both self-report questionnaires and accelerometer measurements. For this objective, in cases where accelerometer cutoff values calibrated by a specialized lab are not available, we recommend drawing on both questionnaire-based and accelerometer-based data, rather than prioritizing one over the other. Although questionnaires inherently include subjective noise, the averaging process can mitigate some of its effects, and standardized questionnaire formats are recognized and used worldwide. By contrast, accelerometer data may introduce direct bias in PA adherence rates unless cutoff values appropriate to the population are applied. Therefore, in absence of a laboratory-calibrated cutoff threshold, accelerometer data do not necessarily hold an absolute advantage over questionnaire data. We also do not endorse any specific accelerometer cutoff value simply because it yields, on average, results comparable to those obtained from questionnaire data.*

*On the other hand, for the second objective—investigating factors associated with PA adherence using both measurement tools—we recommend placing greater emphasis on accelerometer data. In our correlates analysis, we found that subjectivity issues directly affect the interpretation of the variables; therefore, accelerometer data offer an absolute advantage in this context.*

**19. Lines 339 – 341: Could you provide explanations or hypotheses regarding the association with these health factors?**

**Our Response:**

Thank you for your comment. Following the reviewer’s suggestion, we have added the following proposals for potential hypotheses regarding these variables to the revised manuscript:

- Lines 446-453: *Interestingly, health conditions, such as diabetes, hypertriglyceridemia, and low HDL cholesterol levels, were also found to be significant factors. It should be noted that the significant covariates obtained from observational studies only guarantee correlation rather than causation, so caution is warranted in their interpretation. One possible explanation or hypothesis is that non-adherence to PA guidelines may have led to these significant outcomes (i.e., diabetes, hypertriglyceridemia, and low HDL cholesterol). In other words, one could interpret that non-adherence has, in turn, influenced the covariates, resulting in the observed significant correlations. To infer causation, randomized controlled trials are essential.*

**20. Lines 342, 343: As highlighted in a comment about the introduction content, we could understand that using questionnaires is a gold-standard. Please rephrase.**

**Our Response:**

Thank you for your comment. In line with the reviewer’s suggestion, to avoid any misconception that the questionnaire is the gold standard, we have rephrased the sentence in the revised manuscript as follows:

- Lines 463-467: *Notably, the findings from the Bland-Altman plots demonstrate that two scenarios (i.e., b and c, which reflect a 1952 cpm 10-minute bout with a 1–2-minute tolerance and a 1040 cpm 10-minute bout, respectively) show minimal bias when comparing self-reported PA with objective measures. However, it should be noted that the questionnaire method is limited by respondents’ subjectivity and therefore cannot serve as a standard for accelerometer cutoff values.*

**21. Line 350 – 352: What evidence is there to support this assertion?**

**Our Response:**

Thank you for your comment. As noted in our responses to Comments 1, 5, and 17, the assertion regarding Line 350 – 352 has been withdrawn. We do not endorse any single threshold as preferable for Korean older adults, which should clarify that questionnaire data are not being treated as a gold standard. In accordance with the reviewer’s suggestion, we have removed this argument from the revised manuscript.

**22. Line 350 – 358: What are your recommendations to readers? Which method is the best (threshold, with without tolerance)? How your results can support it?**

**Our Response:**

Thank you for your comment. As noted in our responses to Comments 1, 5, and 17, the assertion regarding Line 350 – 358 has been withdrawn. We do not endorse any single threshold as preferable for Korean older adults, which should clarify that questionnaire data are not being treated as a gold standard. In accordance with the reviewer’s suggestion, we have removed this argument from the revised manuscript.

**23. Line 358: This may partly explain the discrepancies observed but did you get the information about the participant engagement in water-based physical activities?**

**Our Response:**

We appreciate the reviewer’s concern that mentioning unlikely situations in our dataset could potentially confuse readers. In fact, the guidelines provided by the Korea Disease Control and Prevention Agency indicate that, although the Actigraph GT3X+ model is waterproof, participants were instructed to remove the device to ensure device safety when engaging in activities such as swimming or bathing [36]. This is stated in Lines 109-110 of the original manuscript (Lines 148-149 in the revised version). Therefore, we have decided to remove the statement in Line 358, as these scenarios are unlikely to have a significant impact on our dataset. Thank you for your thorough review.

Reference:

[36] Korea Disease Control and Prevention Agency. Guidelines for using raw data from the KNHANES accelerometer survey VII 2017. 2018. Available: https://knhanes.kdca.go.kr/knhanes/eng/main.do

**24. Conclusion**

**Line 380, 381: What conditions enabled to get a minimal bias? This sentence is probably not appropriate since you did not compare biases according to factors but you compared the alignment according to algorithm parameters (thresholds) to detect MVPA. This should be rephrased.**

**Our Response:**

Thank you for your comment. As noted in our responses to Comments #1, #5, and #17, the assertion regarding Lines 380–381 has been withdrawn. We do not endorse any single threshold as preferable for Korean older adults, thereby clarifying that questionnaire data are not being treated as a gold standard. In accordance with the reviewer’s suggestion, we have removed the statement and further clarified the main objectives and conclusions of our study as follows:

- Lines 495-506: *We observed significant demographic variations in PA guideline adherence, including sex, age, hypertriglyceridemia, and low HDL cholesterol levels. However, our study discovered discrepancies between questionnaire data and accelerometer data in analyzing the key factors associated with PA adherence, demonstrating that the potential subjectivity in questionnaire data may introduce issues in interpreting covariates.*

*From the standpoint of policy formulation, it is crucial to rely on objective evidence when assessing PA adherence and identifying its associated covariates. Based on our findings, in evaluating PA adherence rates among the target population (in this case, older adults in Korea), we recommend treating both questionnaire and accelerometer data as equally valid sources, since data reflecting diverse perspectives are essential for developing effective public policies. Meanwhile, we suggest prioritizing accelerometer data when exploring the correlates of PA adherence, given that the inherent subjectivity of questionnaire-based measures more directly affects their interpretation.*

**25. Figures**

**Figure 2 and Figure 3: Please specify the units on the axes.**

**Our Response:**

Thank you for the comment. Following the reviwer’s suggestion, we have added the units to the axes. Because the accelerometer data only accounts for valid wear days, we used minutes per day as to indicate the duration of activity. Also, there are now a total of four Bland-Altman plots addressing all four reference-based scenarios (mentioned in Comment #16) as follows:

(a) 1952 cpm 10-minute bout,
(b) 1952 cpm 10-minute bout with a 1–2-minute tolerance,
(c) 1040 cpm 10-minute bout, and
(d) 1040 cpm 10-minute bout with a 1–2-minute tolerance.

We hope these revisions address your concerns and improve the clarity of our presentation.

- Figure 2:

**26. I find the shapes of the plots are a bit strange because of the linear pattern (like a triangle). Is there an explanation about these shapes?**

**Our Response:**

Thank you for your observation regarding the seemingly linear (triangular) pattern in the Bland-Altman plots. We agree that at first glance, these shapes may appear unusual, and we would like to clarify that this pattern indicates that the two methods are extremely inconsistent:

1. Threshold-based binary-style classification

In accelerometer data, MVPA minutes per day are calculated based on specific cut points (e.g., 1952 cpm or 1040 cpm) and classified as either ‘bout’ (meeting a minimum duration threshold) or ‘non-bout.’ The total bout time tends to increase when a 1–2-minute tolerance is allowed. Such threshold-based approaches can yield extreme values (i.e., zero), leading to clusters of data points. This phenomenon also occurs when respondents fill out questionnaires, and these extreme values contribute to the triangular pattern observed in the Bland-Altman plot. When plotted as difference versus average in the Bland-Altman framework, these extreme values can create visually distinct, wedge- or triangle-like patterns.

2. Linear (triangular) pattern in the Bland-Altman plots

Both self-reported and accelerometer-measured MVPA minutes/day are inherently bounded at the lower end (0 minutes) and have practical upper limits. In the Bland-Altman plot, the vertical axis represents the difference between the two measurements, and this difference is maximized when one of them is zero. For example, when 𝑥=𝑎 and 𝑦=0, then the horizontal axis is (𝑥+𝑦)/2 = 𝑎/2 and the vertical axis is 𝑥-𝑦 = 𝑎. As the average increases along the x-axis, the potential range for the difference on the y-axis expands, forming a triangular contour.

3. Contribution of Zero Values

Participants reporting 0 minutes in both self-reported and accelerometer-based measures cluster at the origin (0,0), causing a concentration of data points in that corner of the plot. The data points along the upper line represent participants who reported a high level of PA in the questionnaire but showed no recorded activity on the accelerometer (i.e., extreme over-reporters), while the data points along the lower line represent participants who did not report any PA in the questionnaire but had activity recorded by the accelerometer (i.e., extreme under-reporters). Meanwhile, when both measurements are non-zero and exhibit a difference, the data points appropriately spread within the triangular region according to their distribution, and if the two measurements match exactly, the value on the vertical axis coincides with zero.

A triangular-shaped Bland-Altman plot suggests that the data are non-negative and that there are many instances in which one of the measurements is zero (note that the data represent MVPA minutes rather than an adherence rate). This shape highlights the unique distribution of our data and the influence of threshold-based measurement. The value labeled ‘Mu_difference’ in the Bland-Altman plots represents the average difference—i.e., the bias—between the two measurements. As the reviewer suggested, it should be interpreted as the difference between the two measurements only, and should not be used as an absolute measure indicating that the questionnaire data are the gold standard.

We hope this clarifies why our Bland-Altman plots exhibit the triangular pattern. Thank you again for your thoughtful comment and for giving us the opportunity to further explain these findings. With respect to the point raised by the reviwer, we revised the manuscript as follows:

- Lines 359-378: *Fig 2 displays Bland-Altman plots for daily MVPA minutes comparing the questionnaire (Q = K-GPAQ) and the accelerometer under four different scenarios: panel A uses a >1952 cpm cutoff (A1952); panel B applies a >1952 cpm cutoff with a 1–2‐minute tolerance (A1952t); panel C uses a >1040 cpm cutoff (A1040); and panel D applies a >1040 cpm cutoff with tolerance (A1040t). The horizontal axis represents the mean of the two measures, as defined in Equation (1), while the vertical axis shows their difference, as specified in Equation (2). In each plot, the dashed central line represents the average difference (bias) across all participants, and the solid lines represent the 95% limits of agreement (± 1.96 SD), calculated using Equation (3). A narrower spread between the solid lines indicates closer agreement, whereas a wider spread suggests greater variability between methods.*

***Fig 2. Bland-Altman plots for daily MVPA minutes comparing the questionnaire data (Q = K-GPAQ) with accelerometer data under the following settings: A. A1952 (>1952cpm). B. A1952t (>1952cpm, tolerated). C. A1040 (>1040cpm). D. A1040t (>1040cpm, tolerated).***

*Panels A and B—which use a higher cutoff (>1952 cpm)—show average differences of 13.81 and 6.72 minutes/day, respectively, with the questionnaire reporting higher MVPA times. In contrast, panels C and D—which use a lower cutoff (>1040 cpm)—exhibit average differences of 2.34 and -15.77 minutes/day, respectively. This implies that applying a higher cutoff criterion for determining 10-minute bouts in the accelerometer data results in a lower recorded MVPA time. Moreover, the mean difference is numerically lower when applying a 1–2 minute tolerance: panel B shows a lower difference than panel A, and panel D a lower difference than panel C. This suggests that even with the same cutoff, incorporating a 1–2 minute tolerance in bout determination tends to increase the recorded MVPA time, as if the cutoff were effectively lowered. Although one combination (panel C, >1040 cpm) exhibited a relatively small overall bias, the triangular boundaries observed in the Bland-Altman plots indicate a pronounced discrepancy between the questionnaire and accelerometer data. These triangular patterns represent cases where one method recorded 0 minutes/day of MVPA: points along the upper boundary correspond to instances where MVPA was reported in the questionnaire but not detected by the accelerometer, whereas points along the lower boundary indicate cases where MVPA was detected by the accelerometer but not reported in the questionnaire.*
